# The conjugation-resistant bile acid norUDCA cures liver fibrosis but impairs systemic energy metabolism

**DOI:** 10.1016/j.molmet.2026.102363

**Published:** 2026-04-02

**Authors:** Ioannis Evangelakos, Esther Verkade, Julia K. Rohde, Alex Zaufel, Martin Vargek, Markus Heine, Anna Worthmann, Sebastian Graute, Marceline Manka Fuh, Karthikeyan Gunasekaran, Manju Kumari, Dorothee Schwinge, Martin von Bergen, Ulrike Rolle-Kampczyk, Beatrice Engelmann, Rolf Breinbauer, Rita Fuerst, Umber Saleem, Jan Freark de Boer, Christian Schlein, Arne Hansen, Ludger Scheja, Folkert Kuipers, Tarek Moustafa, Joerg Heeren

**Affiliations:** 1 Department of Biochemistry and Molecular Cell Biology, University Medical Center Hamburg-Eppendorf, Hamburg, Germany; 2 Institute of Human Genetics, University Medical Center Hamburg-Eppendorf, Hamburg, Germany; 3 Department of Pediatrics, University of Groningen, University Medical Center Groningen, Groningen, the Netherlands; 4 Division of Gastroenterology and Hepatology, Department of Internal Medicine, Medical University of Graz, Austria; 5 Department of Medicine I, University Medical Center Hamburg-Eppendorf, Hamburg, Germany; 6 Department of Molecular Systems Biology, Helmholtz Centre for Environmental Research, Leipzig, Germany; 7 Institute of Organic Chemistry, Graz University of Technology, Stremayrgasse 9, Graz, 8010, Austria; 8 Institute of Experimental Pharmacology and Toxicology, University Medical Center Hamburg-Eppendorf, Hamburg, Germany; 9 European Research Institute for the Biology of Ageing (ERIBA), University of Groningen, University Medical Center Groningen, Groningen, the Netherlands

**Keywords:** Bile acids, Brown adipose tissue, Energy substrates, Ketone bodies, Glucose metabolism

## Abstract

Bile acids (BAs) play an important role in systemic metabolic improvements following bariatric surgery. In this study, we found that orally administered norursodeoxycholic acid (norUDCA), a conjugation-resistant C23 derivative of naturally occurring UDCA, accumulated in peripheral organs including heart and brown adipose tissue (BAT). Moreover, norUDCA decreased systemic levels of endogenous conjugated BAs, while increasing unconjugated BAs. Notably, in addition to beneficial effects in a cholestatic liver disease model, norUDCA also lowered plasma glucose and fat mass in mice, suggesting that this BA derivative could be repurposed for treating obesity-associated cardiometabolic diseases. Metabolic energy expenditure studies, however, revealed that norUDCA-treated mice have impaired BAT capacity and developed intolerance to cold stress, a phenotype exacerbated in mice lacking adipose ATGL-dependent lipolysis. Transcriptomic and metabolic analyses demonstrated tissue remodeling in heart and BAT that involved pronounced changes in energy substrate utilization, including enhanced cardiac glucose uptake and higher ketone body utilization in BAT. Importantly, co-administration of a low-carb diet prevented cold stress-induced metabolic deficits. Mechanistic studies in human engineered heart tissue indicated that norUDCA compromised contractile function. In conclusion, these data suggest that conjugation-resistant BA derivatives like norUDCA impair myocardial and BAT energetics by altering glucose, lipid, and energy metabolism, particularly during catabolic cold stress conditions.

## Highlights

• norUDCA supplementation significantly alters the systemic bile acid profile
• norUDCA-treated mice are intolerant to cold stress
• norUDCA shifts energy substrate utilization and impairs mitochondrial respiration
• norUDCA causes tissue remodeling in heart and brown adipose tissue

## Graphical abstract

**Figure:**
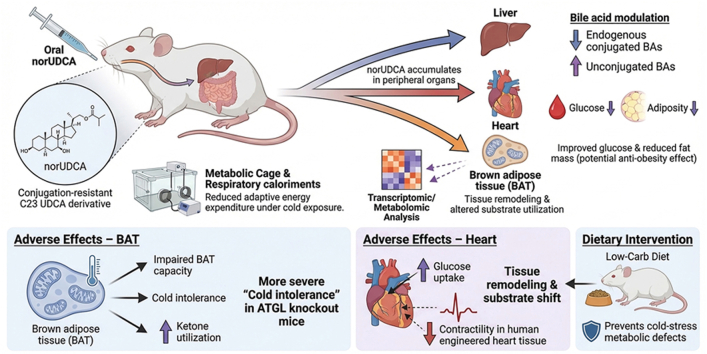

## 1 Introduction

Bile acids (BAs) are cholesterol derivatives that are synthesized in hepatocytes by consecutive steps of enzymatic reactions finalized by conjugation to taurine or glycine. The unconjugated (UBAs) and conjugated (CBAs) species present in the circulating BA pool vary considerably in their biochemical and biophysical properties, which has important physiological and pathological consequences [1]. For example, patients suffering from metabolic dysfunction-associated steatohepatitis, alcoholic liver cirrhosis, or primary biliary cholangitis (PBC) frequently exhibit elevated total BAs, particularly CBAs [2]. These pathological conditions are further characterized by higher CBA/UBA ratios [3], reflecting underlying alterations in the gut microbiome and its capacity for BA-transformation, including the generation of secondary BAs [4]. Supporting a potentially detrimental role of CBAs, a recent study demonstrated that the inhibition of BA conjugation through deletion of the hepatic bile acid–CoA: amino acid N-acyltransferase (BAAT) enhanced tumor-specific T cell responses and reduced tumor growth in liver [5]. In line, conjugation of the gut microbiome-derived secondary BAs 3-oxoLCA and isoalloLCA reduced their immunomodulatory effects on T helper and regulatory T cells [6], indicating that conjugation also of secondary BAs influences adaptive immune responses. Similarly, enriching the BA-pool with hydrophilic BAs, such as ursodeoxycholic acid (UDCA), an approved standard therapy for the treatment of PBC [7], boosts T cell function and consequently abolishes liver injury and concomitant tumors growth in various mouse models [5,[8], [9], [10]]. On the other hand, BA de-conjugation by bacterial bile salt hydrolases (BSHs) in the gut generates UBAs that are less efficient than their conjugated counterparts in the emulsification of dietary lipids and may lead to dysregulated lipid absorption and weight loss [11,12]. Growing evidence suggest that the balance between BA-conjugation and microbial de-conjugation is an important determinant for systemic metabolism, such as during Roux-en-Y gastric bypass (RYGB) surgery. This weight loss procedure causes increased BA levels, predominantly of glycine-conjugated BAs in the postprandial phase [13], which are associated with altered rates of glucose and lipid oxidation [14]. Another example that a higher CBA to UBA ratio regulates systemic energy metabolism is based on the observation that under conditions of BAT activation by cold exposure, adaptive changes in endogenous BA metabolism were associated with efficient heat production as wells as improved lipid and lipoprotein metabolism in mice [15,16]. In another mouse study, focusing on gut microbiome changes in response to cold exposure, the beneficial effects on adiposity and adaptive thermogenesis also involved enhanced CBA production and a higher CBA to UBA ratio [17]. Notably, metabolomics analysis of human plasma samples following BAT activation by the β3-adrenoreceptor agonist mirabegron revealed that BA metabolism was the most affected pathway [18,19].

C23 BAs with a shortened side chain, such as norchenodeoxycholic acid (norCDCA), norcholic acid (norCA) and norUDCA, have been developed to improve therapeutic properties of naturally occurring BAs [20]. These nor-BAs are inefficiently amidated, i.e. they are highly resistant to taurine or glycine conjugation as well as microbial biotransformation. Accordingly, they are secreted in an unconjugated form into bile, which induces hypercholeresis accompanied by increased bicarbonate output [21], thereby alleviating toxic effects particularly of hydrophobic BAs [20]. This is in particular the case for norUDCA, a derivative that combines hydrophilicity with conjugation resistance, and has been successfully used for therapeutic intervention in preclinical models and patients with cholestatic as well as fibrotic liver diseases [[22], [23], [24], [25]]. Moreover, norUDCA has been shown to modulate regulatory networks influencing immune-metabolism of T cells, attenuating T cell-driven inflammatory diseases in gut and liver [26,27]. Importantly, unconjugated norUDCA can reach plasma concentration that are approximately 10-fold higher as compared to other orally administered endogenous BAs including UDCA [26]. However, the consequences of this systemic spill-over for peripheral tissues such as muscle, heart, brown or white fat depots remain elusive.

Given its high bioavailability in the systemic circulation, here we investigated in mice whether norUDCA could serve as a potential and suitable treatment to enhance energy expenditure and thermogenesis. We found that the accumulation of norUDCA in peripheral organs was associated with reduced adiposity and lower blood glucose. Unexpectedly, however, norUDCA treatment resulted in decreased energy expenditure and core body temperature in response to energy-demanding cold exposure. Metabolic tracer and mechanistic studies indicate a critical role of norUDCA for fuel uptake, BAT capacity and whole-body energy metabolism, particularly during adaptive thermogenesis induced by cold exposure. Moreover, our data suggest that elevated systemic levels of norUDCA together with the concomitant increase in endogenous unconjugated BAs cause alterations in glucose and lipid homeostasis that contribute to metabolic inflexibility in cardiac and adipose tissues, impairing their capacity to effectively utilize energy substrates for metabolic processes.

## 2 Results

### 2.1 NorUDCA treatment ameliorates cholestatic liver disease but confers intolerance to cold exposure

**Figure 1:**
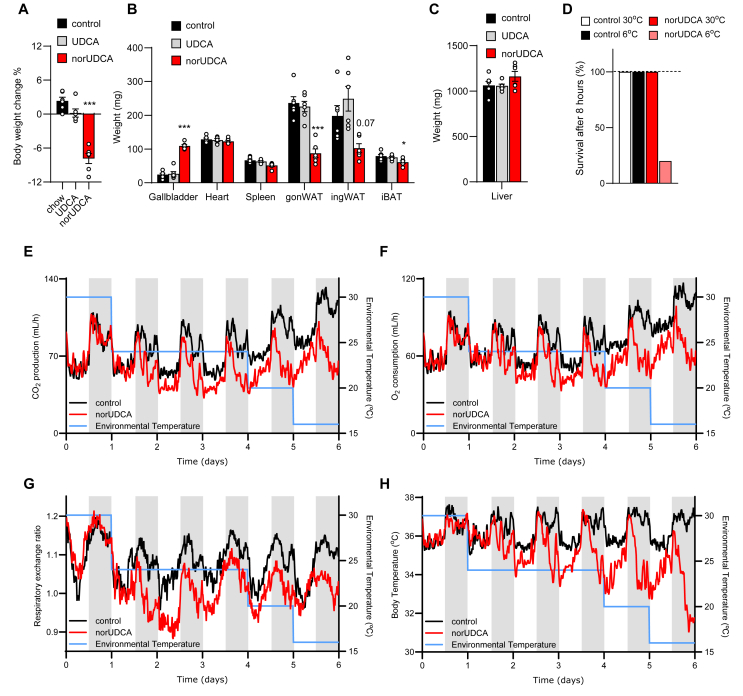
**NorUDCA reduces adipose tissue weights and compromises the thermogenic response to cold exposure. A-C** Wild type mice housed at room temperature were fed a regular chow (control), or chow diet supplemented with either 0.5% UDCA or 0.5% norUDCA. Body weight change (**A**), organ weights (**B**) and liver weights (**C**) after 7 day treatment under standard housing conditions (*n* = *6*). **D** Survival of mice throughout 8 h acclimation to the indicated environmental temperature after feeding a chow diet (control) or a chow diet supplemented with 0.5% norUDCA for 1 week at standard housing temperature (*n* = *8*). **E-H** Indirect calorimetry and body temperature measurements were performed under feeding a chow (control) or norUDCA diet (*n* = *6*). The dietary regimen was started at time point zero and the ambient temperature was gradually reduced as indicated (blue line). CO_2_ production (**E**), O_2_ consumption (**F**), respiratory exchange rate (**G**), body core temperature (**H**) were continuously recorded. Error bars indicate standard error of the mean (SEM). Statistical analysis was performed with one-way ANOVA. ∗p < 0.05, ∗∗p < 0.01,∗∗∗p < 0.001.

To investigate the impact of conjugation-resistance of BAs for systemic energy metabolism, we first compared UDCA versus norUDCA treatment in wild type and *Cyp2c70*^*−/−*^ mice. The latter is a preclinical model with altered bile acid composition leading to concomitant hepatobiliary injury, which is based on the deficient conversion of CDCA into the hydrophilic muricholic acids [28,29]. In the experimental setup, wild type and *Cyp2c70*^*−/−*^ mice were fed a regular chow (control) or diets supplemented with 0.5% of either UDCA or norUDCA, a dosage previously shown to exert therapeutic efficacy on chronic liver injury. Both BAs equally improved the inflammatory and fibrotic liver phenotype in *Cyp2c70*^*−/−*^ mice (Fig. S1A–C), which is in line with a previous study using UDCA [8]. In caecum, norUDCA levels accumulated to a concentration that was 5-fold higher than that of UDCA (Fig. S1D). Notably, norUDCA but not UDCA significantly reduced body weight (Figure 1A), which was mainly explained by reduced fat mass (Fig. S2A), lower weights of adipose tissue depots (Figure 1B) and we observed a minor decrease in food intake (Fig. S2B). Notably, typical fasting-induced hepatic markers, including *angiopoietin-like 4* (*Angptl4*), *pyruvate dehydrogenase kinase 4* (*Pdk4)*, and *acetoacetyl-CoA synthetase* (*Aacs)*, remained unchanged in norUDCA-treated mice housed at an ambient temperature of 22 °C (Fig. S2C–D), indicating that norUDCA supplementation did not mimic a pronounced fasting response. Treatment with norUDCA led to larger gall bladders (Figure 1B, S2E), which was probably a result of hypercholeresis [23,30]. The weights of other organs including liver (Figure 1C) and heart (Figure 1B) were unaffected. As the reduced fat mass suggested enhanced energy expenditure and heat production in BAT and muscle, we determined the effects of norUDCA on adaptive thermogenic responses. To this end, wild type mice fed a normal chow (control) or chow supplemented with norUDCA were housed at thermoneutral conditions (30 °C) or exposed to cold (6 °C). Unexpectedly, the norUDCA-treated mice housed at 6 °C were completely cold-intolerant and 80% died within 8 h of cold exposure (Figure 1D). Furthermore, indirect calorimetry revealed that mice on the norUDCA-supplemented diet exhibited progressively lower respiration rates and body temperatures even when the ambient temperatures were gradually reduced from 30 °C to only 16 °C (Figure 1E–H, S2F–I). Consistent with Fig. S2B, we observed a trend for lower food intake at room temperature in the norUDCA-treated group, while no differences were observed at thermoneutrality (30 °C) or 20 °C (Fig. S2J). At 16 °C, food intake was lower in the norUDCA-treated group. However, given that energy expenditure and core body temperature were already significantly compromised before, the lower food intake likely reflects an impaired feeding behaviour induced by a torpor-like conditions. These data suggest that the reduced fat mass in norUDCA-supplemented mice is not merely a consequence of a hypometabolic state secondary to decreased food intake. Rather, our findings indicate that norUDCA treatment reduces whole-body energy expenditure and thermogenesis, thereby critically impairing survival during cold acclimation.

### 2.2 NorUDCA accumulates in the systemic circulation and peripheral tissues together with endogenous unconjugated BAs

**Figure 2:**
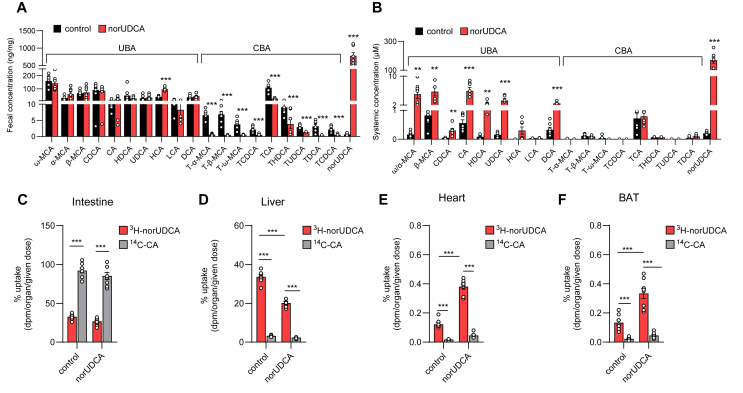
**norUDCA effects on bile acid pool and enrichment in metabolically active organs. A-B** Wild type mice housed at room temperature were fed a regular chow (control), or chow diet supplemented with 0.5% norUDCA. Bile acid concentrations in feces (**A**) and systemic blood plasma (**B**) after 7 day treatment under standard housing conditions (*n* = *7*) **C–F**, Wild type mice were fed a regular chow (control), or chow diet supplemented with 0.5% norUDCA before oral administration of ^3^H-norUDCA and ^14^C-CA (*n* = *8*). Four hours later, percentage of applied tracers was determined in intestine (**C**), liver (**D**), heart (**E**) and BAT (**F**). Statistical analysis was performed with Student's T-Test (**A-B**) or with two-way ANOVA (**C–F**). ∗p < 0.05, ∗∗p < 0.01,∗∗∗p < 0.001.

To determine whether norUDCA exerts its effects on energy expenditure in peripheral organs directly via the circulation, we measured CBA und UBA species in wild type mice fed a normal chow or a norUDCA-supplemented diet that were housed at an ambient housing temperature of 22 °C. Dietary exposure resulted in decreased fecal CBAs and increased total fecal bile acid content, the latter resulting from the exogenous norUDCA (Figure 2A). Despite the high fecal excretion, norUDCA was still significantly enriched in systemic plasma (Figure 2B). Remarkably, concentrations of endogenous UBAs were also increased in plasma with total levels of ∼20 μM. Thus, UBA levels by far exceed those of CBAs with ∼2 μM, indicating a pronounced spillover of endogenous UBAs and norUDCA into the systemic circulation (Figure 2B). To determine the exposure of peripheral organs to norUDCA, we orally administered ^3^H-norUDCA and compared its organ uptake at 4 h with that of simultaneously administered ^14^C-cholic acid (^14^C-CA) in mice fed a chow or a norUDCA-supplemented chow diet. Irrespective of diet composition, almost 90% of radiolabeled CA was detected in the intestine and only minute amounts were found in the liver (Figure 2C). In contrast, only ∼30% of ^3^H-norUDCA ended up in the intestine and a similar amount was found in the liver (Figure 2C, D), which argues for efficient cholehepatic shunting between the bile duct epithelium and liver of norUDCA as compared to CA. Remarkably, a substantial amount of ^3^H-norUDCA compared to ^14^C-CA was detected in peripheral tissues such as heart and BAT, an effect that was even more pronounced in norUDCA-preconditioned mice (Figure 2E–F, S3A-D). Based on a measured systemic concentration of approximately 200 μM (Figure 2B) and the observation that radiolabeled norUDCA levels in the heart and BAT are approximately 10-fold lower than in plasma (Figure 2B, S3D), we estimate that steady-state intracellular concentrations in these metabolic organs can reach approximately 20 μM. These data demonstrate considerable exposure of key metabolic tissues to circulating norUDCA and other UBAs.

### 2.3 NorUDCA induces tissue remodeling and alters energy substrate utilization in heart and BAT

**Figure 3:**
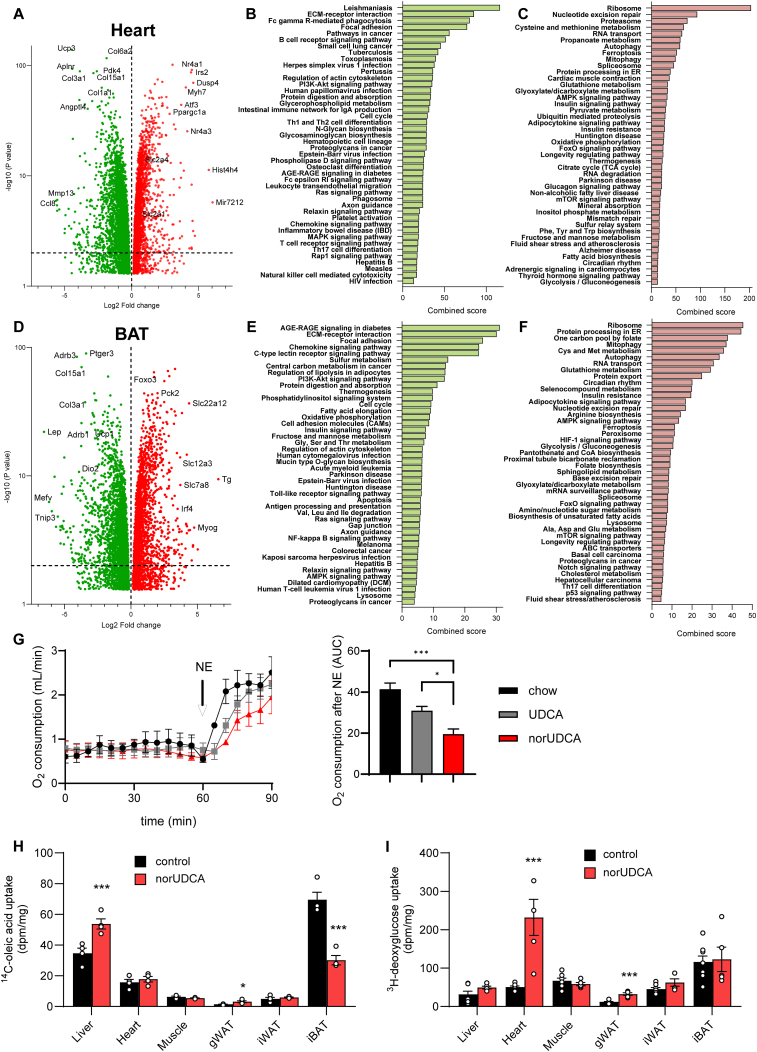
**norUDCA drives transcriptional remodelling and alters energy source utilization in heart and brown adipose tissue. A-H** Wild type mice were fed a regular chow (control), or chow diet supplemented with 0.5% norUDCA for 7 days with housing at room temperature. Bulk RNAseq (*n* = *4*) of heart (**A-C**) and BAT (**D-F**) were performed to identify differentially expressed genes (**A, D**), and to characterize downregulated (**B, E**) and upregulated (**C, F**) pathways determined by gene ontology analysis. **G** Wild type mice were pair-fed a regular chow (control), or chow diet supplemented with 0.5% UDCA or 0.5% norUDCA for 7 days with housing at thermoneutrality. BAT capacity was determined by indirect calorimetry in response to 100 nM norepinephrine injection (n = 5). For quantification, right panel shows quantification by area under curve. Error bars indicate standard error of the mean (SEM). Statistical analysis was performed by one-way ANOVA. ∗p < 0.05, ∗∗∗p < 0.001. **H–I**, Organ-specific uptake of intravenously administered albumin-bound ^14^C-oleic acid (*n* = *4*) (**H**) and ^3^H-deoxyglucose (*n* = *5-8*) (**I**) was determined 15 min after injection. Error bars indicate standard error of the mean (SEM). Statistical analysis was performed with Student's T-Test. ∗p < 0.05, ∗∗p < 0.01,∗∗∗p < 0.001.

Metabolic functions of BAT and heart are critical for physiological responses to cold stress by increasing heat production and blood flow, respectively [31]. To understand the effects of norUDCA in an unbiased manner, bulk RNA sequencing of heart (Figure 3A–C) and BAT (Figure 3D–F) from mice on regular chow or on norUDCA-supplemented chow was performed. Volcano plot analysis indicated a high number of differentially expressed genes (DEG) between the groups both in heart (Figure 3A, Supplementary Table 1) and BAT (Figure 3D, Supplementary Table 2). In hearts, top upregulated genes upon norUDCA feeding were related to ventricular remodeling such as *Atf3* and *Myh7*, as well as those mediating mitochondrial biogenesis like *Ppargc1a* (Figure 3A). Furthermore, *Slc2a1* and *Slc2a4* encoding the glucose transporters GLUT1 and GLUT4, respectively, were upregulated whereas *Pdk4,* known to inhibit pyruvate dehydrogenase, was reduced. The changes in expression of a number of metabolic genes were confirmed by qPCR in an independent study (Fig. S4A). Gene ontology (GO) analysis of DEG data revealed that a few metabolic pathways were downregulated (Figure 3B). On the other hand, pathways related to glucose metabolism, e.g., pyruvate metabolism, glycolysis and gluconeogenesis, as well as tissue remodeling pathways including proteasome, ferroptosis and mitophagy were upregulated in the hearts (Figure 3C). In BAT, the expression of key thermogenic markers including *Ucp1*, *Dio2* and *Adrb3* was downregulated (Figure 3D, Fig. S4B). Consistently, pathways related to core thermogenic functions such as oxidative phosphorylation, fatty acid metabolism and thermogenesis were suppressed in the norUDCA-treated group (Figure 3E). In line, qPCR showed altered expression of lipogenic genes such as *Fasn* and *Scd1* (Fig. S4C). Similar to the findings in heart, pathways related to tissue remodeling were upregulated in BAT of norUDCA-treated mice (Figure 3F). To study the functional implications for BAT-dependent thermogenesis indirect calorimetry was performed in control, UDCA- and norUDCA-treated mice. To this end, mice were pair-fed and housed at thermoneutrality to prevent thermogenic responses through the sympathetic nervous system. In comparison to the chow control and UDCA-treated mice, the oxygen consumption was markedly decreased in norUDCA-treated mice in response to norepinephrine injection (Figure 3G), indicating that norUDCA supplementation diminish BAT thermogenic capacity. Moreover, alterations in metabolic pathways detected by expression analysis were confirmed by radioactive tracer studies showing impaired fatty acid (Figure 3H) but unaltered glucose uptake (Figure 3I) by BAT. Despite compromised lipid uptake, a BAT-specific increase in proteins promoting intravascular processing of triglyceride-rich lipoproteins (GPIHBP1, LPL) and fatty acid uptake (CD36) was observed (Fig. S4D–G), suggesting an induction of compensatory yet futile mechanisms to replenish energy stores in thermogenic adipose tissues of norUDCA-treated mice. In the heart, norUDCA caused a several-fold increase in the uptake of glucose (Figure 3H), while the uptake of fatty acids (Figure 3G) was preserved. This observation, in accordance with the increased expression of genes regulating glucose transport and metabolism (Figure 3A–C), point toward an increased reliance of the heart on glucose as energy source and impaired fatty acid utilization in BAT in response to norUDCA supplementation.

### 2.4 NorUDCA shifts cardiac energy metabolism towards glucose utilization

**Figure 4:**
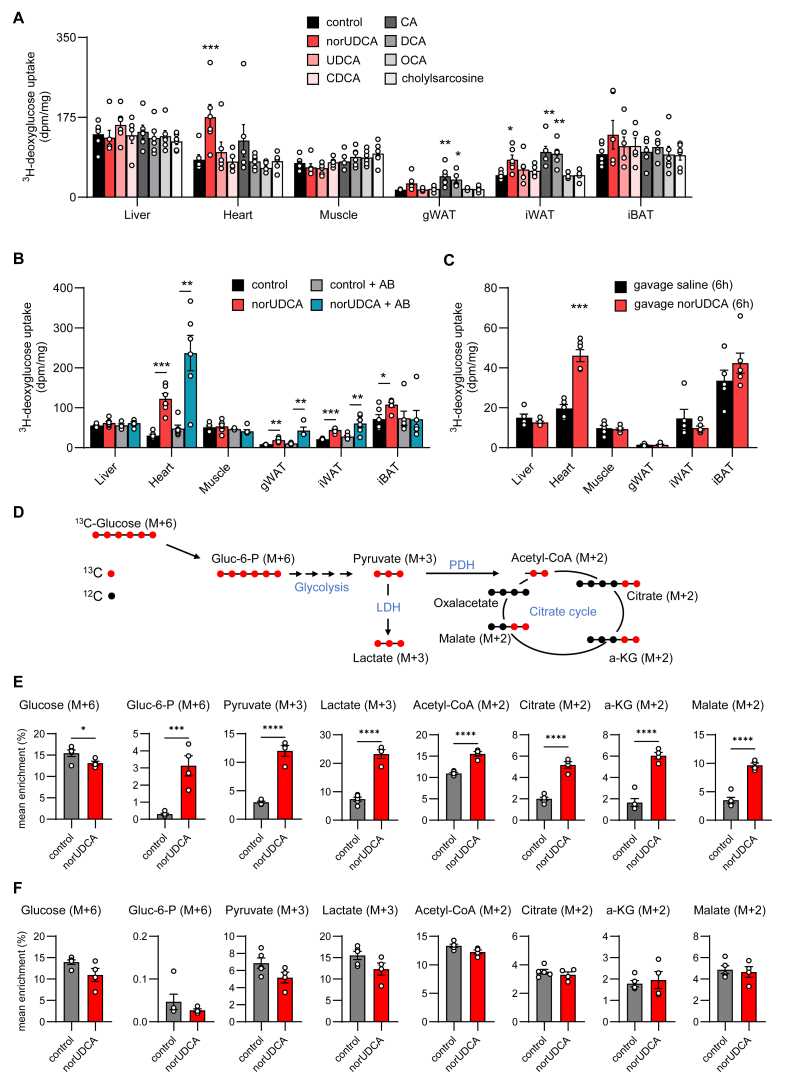
**norUDCA shifts cardiac energy metabolism towards glucose utilization. A** Mice were fed a regular chow (control), or chow diet supplemented with various bile acids at 0.5% (except OCA, 0.05%) for 7 days with housing at room temperature. Organ-specific uptake of intravenously administered ^3^H-deoxyglucose (*n* = *5-6*). **B,** Mice were treated without or with an antibiotic cocktail to deplete gut bacteria and fed a regular chow (control) or a chow diet supplemented with norUDCA for 7 days at room temperature. Organ-specific uptake of ^3^H-deoxyglucose was determined 15 min after intravenous administration (*n* = *6*). **C**, Saline or norUDCA were administered by oral gavage. Organ-specific uptake of ^3^H-deoxyglucose was determined 15 min after intravenous administration 8 h after gavage. **D-F**, Mice were fed a regular chow (control), or chow diet supplemented with norUDCA at 0.5% for 7 days with housing at room temperature. Subsequently, ^13^C-(U) glucose was intravenously injected and 30 min later organs for metabolite enrichment analysis (*n* = *4-5*). Schematic model showing *in vivo* stable isotope labelling of glycolysis and citric acid cycle (**D**) that were quantified in heart (**E**) and BAT (**F**) to determine ^13^C enrichment in metabolites. Error bars are shown as SEM. Statistical analysis was performed either with one-way ANOVA (**A**), two-way ANOVA (**B**) or Student's T-Test (**C, E, F**). ∗p < 0.05, ∗∗p < 0.01,∗∗∗p < 0.005, ∗∗∗∗p < 0.001.

Given the relevance of alterations in glucose metabolism in the context of heart failure [32], the specificity of norUDCA on cardiac glucose uptake compared to other bile acids was determined. For this purpose, ^3^H-deoxyglucose was injected into wild type mice which were fed a chow diet supplemented with norUDCA, as well as endogenous UBAs such as UDCA, CDCA, CA, the secondary bile acid deoxycholic acid (DCA), obeticholic acid (OCA, a semi-synthetic bile acid analogue which has the chemical structure 6α-ethyl-CDCA), as well as cholylsarcosine, the synthetic conjugate of cholic acid and sarcosine, which is resistant to de-conjugation during enterohepatic cycling. All UBAs were applied in a g/mol range (norUDCA 378.5 g/mol UDCA, CDCA, DCA ∼392.6 g/mol; CA ∼408.6 g/mol) to produce similar systemic exposure, with the exception of semi-synthetic conjugated BA, cholylsarcosine 479.6 g/mol and OCA at (0.05% w/w), acting as a potent FXR agonist). Among all the bile acid species tested, only norUDCA significantly increased ^3^H-deoxyglucose uptake in the heart (Figure 4A). Supplementation with norUDCA but also with CA and DCA resulted in higher glucose uptake into white adipose tissue depots, while no significant changes were detected in other organs investigated (Figure 4A). The effect of norUDCA on cardiac glucose disposal was also observed in the FVB mouse strain, hyperlipidemic *Apoa5*-deficient FVB and *Cyp2c70*^−/−^ mice (Fig. S4H–I). We next determined whether the microbiome is involved in mediating the effect of norUDCA on shifting energy substrate utilization. For this purpose, we depleted the gut bacteria by antibiotic treatment (Abx), known to decrease the turnover of bile acids and to elevate plasma levels of CBAs [33]. Of note, the heart of norUDCA-treated mice show a tendency to even higher ^3^H-deoxyglucose uptake after Abx treatment (Figure 4B), indicating that microbial processing is not essential for the observed norUDCA effect on glucose metabolism. Of note, already 6 h after oral administration of norUDCA the heart internalized larger amounts of ^3^H-deoxyglucose (Figure 4C), indicating an acute effect that is independent of processes induced by chronic administration, e.g. loss in fat mass or altered caloric intake. Next, we performed metabolic flux studies to determine how norUDCA and the associated higher glucose uptake impacts intracellular glucose metabolism. To achieve this goal, control and norUDCA-fed mice received an intravenous bolus of ^13^C-labeleled glucose and enrichment of ^13^C in glucose metabolites (schematic diagram in Figure 4D) was assessed by mass spectrometry in heart (Figure 4E) and BAT (Figure 4F). In line with higher glucose uptake, norUDCA treatment caused a markedly increase in glucose metabolism as indicated by higher enrichments of ^13^C in intermediates of glycolysis including lactate and citrate cycle in heart (Figure 4E) but not in BAT (Figure 4F). Overall, cardiac tissue of mice treated with norUDCA exhibits a profound increase in glucose uptake and utilization, a phenotype often related to heart failure and cardiac hypertrophy [32].

### 2.5 Lipolysis and ketone bodies compensate norUDCA-induced defects in energy homeostasis and thermogenesis

**Figure 5:**
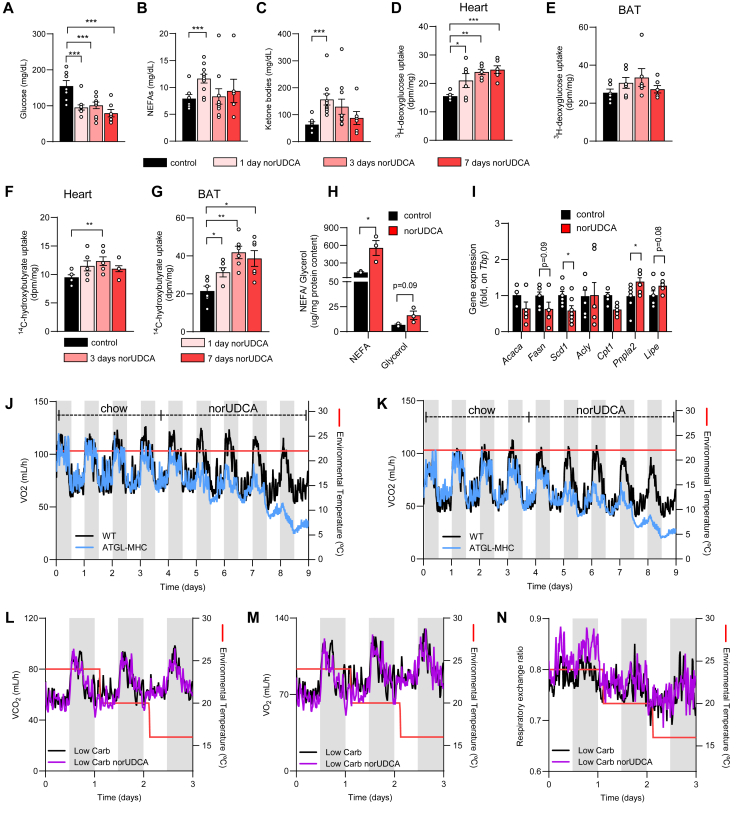
**Lipolysis and ketone bodies compensate norUDCA-induced defects in energy homeostasis and thermogenesis. A-G** Mice were fed a regular chow (control), or chow diet supplemented with norUDCA for 1 day, 3 days or 7 days with housing at room temperature. **A-C** Circulating levels of glucose (**A**), non esterified fatty acids (NEFA) (**B**), and ketone bodies (**C**) were determined (*n* = *7*). **D-G**, Uptake of ^3^H-deoxyglucose (**D, E**) and ^14^C-hydroxybutyrate (**F, G**) by heart (**D, F**) and by BAT (**E, G**) was determined 15 min after intravenous administration (*n* = *6*). **H** Release of NEFA and glycerol from white adipose tissue explants isolated from mice fed a chow (control) or chow diet supplemented with norUDCA (*n* = *3*). **I**, Expression of lipogenic and lipolytic genes in white adipose tissues isolated from control and norUDCA-fed mice (*n* = *7*). **J-K** Indirect calorimetry was performed at room temperature in lipolysis-deficient ATGL-MHC and control mice fed a chow for 3 days followed by feeding a norUDCA-containing chow diet for 5 days (*n* = *2-3*). Consumption of O_2_ (**J**) and production of CO_2_ (**K**) are shown. **L-N** Indirect calorimetry was performed in wild type mice that were fed a low-carb diet or low-carb diet supplemented with norUDCA. Production of CO_2_ (**L**), consumption of O_2_ (**M**) and respiratory exchange ratio (**N**) were recorded at various housing temperatures as indicated by the red line (*n* = *3*). Error bars are shown as SEM. Statistical analysis was performed with one-way ANOVA (**A-G**), or Student's T-Test (**H–I**). ∗p < 0.05, ∗∗p < 0.01,∗∗∗p < 0.001.

Normal cardiac function relies on the continuous supply of fatty acids as energy substrate [32]. These free acids delivered to the heart are derived from either adipose tissue lipolysis and/or hydrolysis of triglyceride-rich lipoproteins by lipoprotein lipase [34]. To further unravel disturbances in energy substrate utilization, plasma levels of glucose, non-esterified fatty acids (NEFAs) and ketone bodies were quantified in mice fed with norUDCA for 1 day, 3 days and 7 days, respectively (Figure 5A–C). Glucose levels were strongly reduced at day 1 and remained at lower levels on the following days, which coincides with increased uptake by heart (Figure 5D) but not by BAT (Figure 5E). In contrast, NEFAs and ketone bodies were increased in plasma of norUDCA-treated mice (Figure 5B–C), which was accompanied by a marked increase in the uptake of the ketone body β-hydroxybutyrate in BAT and to a lesser extent by the heart (Figure 5F–G). Consistently, the expression of β-hydroxybutyrate dehydrogenase 1 (*Bdh1*), the rate-limiting enzyme for ketone body utilization in peripheral tissues, is higher in norUDCA-treated mice (Fig. S4B).

The higher levels of ketone bodies are probably triggered by an increased flow of fatty acids from white adipose tissues to liver, as supported by increased lipolysis in adipose tissue explants of norUDCA-treated mice (Figure 5H). In line, higher expression of adipose tissue triglyceride lipase (ATGL) encoded by *Pnpla2* was found in WAT of norUDCA-treated mice (Figure 5I). To directly study the effect of ATGL, we performed indirect calorimetry studies in whole body *Pnpla2* deficient mice with transgenic cardiac ATGL overexpression, which prevents lipid accumulation and heart dysfunction described for the whole body knockout mice [35]. Of note, these mice lacking ATGL in adipose tissues displayed reduced energy expenditure even at 22 °C after norUDCA treatment (Figure 5J–K, S5A-D). Importantly, applying a low carb dietary regime to mice during norUDCA supplementation completely omitted progressively lower respiration rates when the ambient temperature was gradually decreased to 16 °C (Figure 5L-N). Overall, these data indicate that adipose ATGL-mediated lipolysis and subsequent hepatic ketone body production are critical in maintaining energy homeostasis in norUDCA-treated mice. Moreover, dietary approaches allow to overcome disturbed energy substrate utilization in norUDCA fed animals, thereby preventing cold stress-induced metabolic deficits.

### 2.6 NorUDCA impairs mitochondrial respiration and disturbs contractile function of human engineered heart tissue

**Figure 6:**
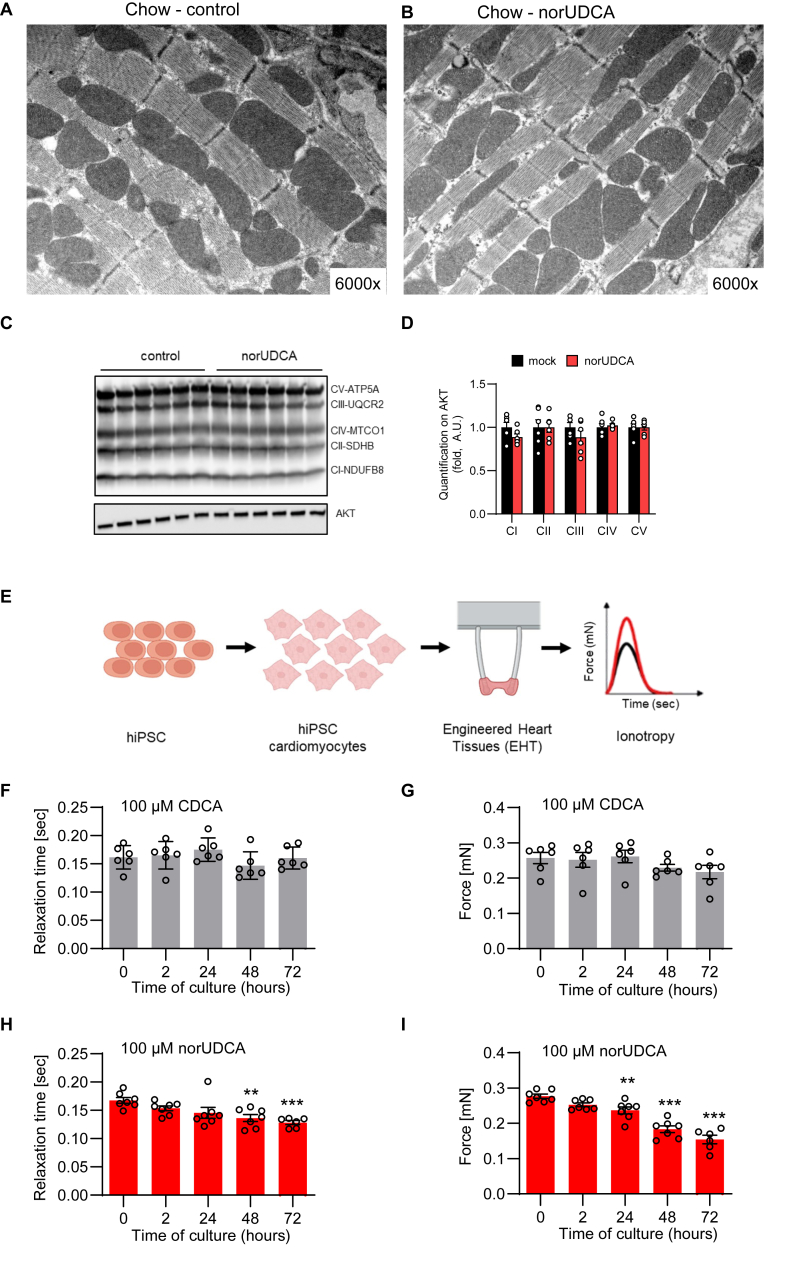
**norUDCA deteriorates mitochondrial respiration and causes functional defects in human engineered heart tissues. A-E**, Mice were fed a regular chow (control), or chow diet supplemented with norUDCA for 7 days with housing at room temperature. Representative electron microscopy pictures of heart from control (**A**) and norUDCA-treated mice (**B**).Western blot analysis of OXPHOS protein complexes from cardiac tissues (**C, D**). **E-I** As depicted in the schematic model (**E**), engineered heart tissues (EHT) were generated from human induced pluripotent stem cells (hiPSCs). **F–I**, EHT were incubated with CDCA (**F, G**) or norUDCA (**H, I**), and relaxation time (**F, H**) and force (**G, I**) were measured at indicated time points (*n* = *6-7*). Error bars are shown as SEM. Statistical analysis was performed either with Student's T-Test (**D**) or one-way ANOVA (**F–I**). ∗p < 0.05, ∗∗p < 0.01,∗∗∗p < 0.001.

The profound increase in cardiac glucose uptake and lactate production suggested a negative effect of norUDCA on mitochondrial function. Surprisingly, the overall architecture of cardiomyocytes and mitochondrial structure visualized by electron microscopy were not conspicuously altered by norUDCA treatment (Figure 6A–B). Similarly, no morphological changes were observed in thermogenic adipocytes and mitochondria of BAT (Fig. S6A–B). In addition, the levels of selected OXPHOS complex proteins were comparably abundant in heart mitochondria isolated from control and norUDCA-treated mice (Figure 6C–D). To test the impact of norUDCA in a relatively integrated cellular system, we incubated engineered heart tissues generated from human induced pluripotent stem cells (see model in Figure 6E) with norUDCA or CDCA. Of note, norUDCA impaired both relaxation time and force generation gradually during incubation, which was not observed using equal concentrations of unconjugated CDCA (Figure 6F–I). Moreover, compared to untreated controls, we observed a dose-dependent effect for norUDCA but not CDCA on force generation (Fig. S6C–D). Taken together, these data indicate that norUDCA impairs cardiomyocyte function in human models systems *in vitro*.

## 3 Discussion

Despite recent progress in understanding the structural diversity and the specific functions of individual BAs, their divergence in the capacity to regulate hepatic and systemic metabolic adaptations remains underexplored. Given the known role of endogenous BAs in stimulating energy expenditure via the G-protein coupled receptor TGR5 in skeletal muscle and BAT both in mice and humans [[36], [37], [38], [39]], a major goal of this study was to investigate the potential beneficial effects of conjugation-resistant norUDCA, a C23-norderivative of UDCA, on systemic energy metabolism. Next to its known anti-fibrotic effects in the hepatobiliary system, its higher systemic concentration compared to naturally occurring bile acids makes norUDCA an attractive candidate to treat obesity-associated comorbidities such as dyslipidemia, diabetes and cardiovascular disease [1,40]. In the current study, we confirmed and complement previous reports that modifying the BA-pool towards higher hydrophilicity with UDCA or norUDCA completely prevents liver inflammation and fibrosis in *Cyp2c70*^−/−^ mice, a mouse model with characteristics of human cholestasis [28]. In metabolic turnover studies, we found a marked enrichment of norUDCA in peripheral organs that was associated with changes in energy substrate utilization in heart and BAT. Against our expectation that norUDCA would be pro-thermogenic, we observed that norUDCA caused a drastic drop in whole body energy expenditure and thermogenesis under catabolic conditions triggered by cold stress. Importantly, the observed metabolic changes/adaptions during the first week of norUDCA supplementation seem not to compromise the vital functions of animals at room temperature, but deteriorates during cold exposure, causing decompensation of systemic energy homeostasis and death of the animals. The slight decrease in food intake observed in norUDCA-supplemented mice may be attributed to the anorexigenic effects of bile acids, which are typically mediated by TGR5-stimulated secretion of glucagon-like peptide-1 and peptide YY [41]. Although norUDCA does not directly bind to TGR5 [42], the norUDCA-induced shifts in the endogenous bile acid pool may indirectly enhance the secretion of these satiety hormones from enteroendocrine L-cells explaining the slightly reduced food intake at room temperature.

Notably, uncoupled respiration for heat production in BAT is mediated by elevated CBAs rather than UBAs [43]. Moreover, beneficial effects of cold on adiposity and adaptive thermogenesis is associated with a higher CBA to UBA ratio [16,17]. Similarly, the metabolic effects of BAs in Roux-en-Y gastric bypass bariatric surgery are related to high levels CBAs and a concomitant decrease in UBA levels [13]. Notably, the phenotype of mice treated with norUDCA shares similarities with that observed after biliopancreatic diversion. The shortened route of enterohepatic circulation in this weight loss intervention characterized by inefficient lipid absorption results in significantly elevated UBAs up to ∼20 μM [44]. The increase in UBA levels in our study could be the result of impaired hepatic conjugation that has been reported to be mediated by C23 nor-bile acid derivatives [45], or result from competition of excessive norUDCA with other UBAs during hepatic extraction from the portal blood [46]. Besides, high UBAs may be a consequence of the activation of microbial bile salt hydrolase (BSH) through norUDCA [47,48], which is mirrored by decreased CBAs in feces found in our study. Based on an estimate of ∼3 g of diet consumed per day per 30 g body weight, the dose of norUDCA was approximately 500 mg/kg/day. Previous studies have proven that norUDCA at this dosage regime improves inflammation and liver injury in various mouse models [49] and even at much higher concentrations (>2500 mg/kg) did not reveal any adverse toxicological effects [50]. In any event, it is very likely that the high ratio of UBA to CBA in plasma provokes the negative effects on energy expenditure and heat production. Still it remains difficult to disentangle direct effects of norUDCA from secondary consequences of altered bile acid pool composition, including changes in BA conjugation and hydrophobicity.

An excess of BAs is known to impair cardiac function [51,52], impact energy metabolism and BAT thermogenesis [43,53], as well as mitochondrial function [54,55]. These effects were observed in both mice and humans with implications for overall energy metabolism and heart function [[56], [57], [58]]. The relationship between “cardiotoxic” BAs and cardiac dysfunction is only witnessed in the context of liver disorders [59,60]. Particularly, hydrophobic BAs with high cellular cytotoxicity mediate such pathological effects *in vitro*, whereas hydrophilic BAs, such as UDCA or muricholic acids even counteracted the effect of lipophilic BAs [61]. NorUDCA that is even more hydrophilic than UDCA (critical micellar concentration is 17 mM for norUDCA versus 7 mM for UDCA; [62]), did even not cause adverse effects when administered to mice during obstructive cholestasis, in whom serum BAs exceed 1000 μM [23]. The uptake, as shown by tracer studies using radiolabeled norUDCA, and in consequence the accumulation of norUDCA within extrahepatic organs could be mediated by the organic anion transporting polypeptide 1 (OATP1), a bile acid transporter that is expressed in multiple tissues. OATP1 has been demonstrated to efficiently transport unconjugated nor bile acids but not other UBAs such as CDCA [63]. Remarkably, the metabolic deficits observed in our study were highly specific for norUDCA, since adverse effects were not observed for closely related UDCA or other BAs. While the other BAs can act as TGR5 or FXR agonist, UDCA is only as weak TGR5 ligand, and norUDCA does not exert any direct effect on TGR5 or FXR [42,49].

It is well established that a dysbalance between glucose and fatty acid oxidation is detrimental for cardiac function [32]. In the current study norUDCA caused hypoglycemia as well as tissue remodeling and inflammation in heart and BAT, which we linked to impaired mitochondrial respiration of palmitic acid and increased flux of cardiac glucose.

Our data indicate that disturbed energy substrate utilization underlies the adverse effects of norUDCA, a notion that is supported by our observation that feeding a low-carb ketogenic diet prevented norUDCA-induced cold intolerance. Ketone bodies are easy to oxidize fuels for peripheral tissues that are produced in the liver from fatty acids released under catabolic conditions by ATGL-mediated lipolysis in the adipose tissues [64]. Notably, cardiac glucose uptake increased >10-fold during acute cold exposure in mice with reduced adipose tissue lipolysis [65] and was associated with pathological remodeling of the heart during chronic cold stress. The relevance of this pathway is highlighted by our observation that mice lacking ATGL in adipose tissues succumb to norUDCA supplementation already at room temperature. Our data therefore suggest that at room temperature, the higher levels of non-esterified fatty acids and ketone bodies can compensate energy disturbances induced by norUDCA supplementation in healthy control animals.

Careful consideration must be given when discussing the potential clinical relevance of our findings regarding translational framing. Importantly, norUDCA can reach significant levels in the systemic circulation in humans, as suggested by a pharmacokinetic study in six healthy male volunteers. These probands were treated with a single oral dose of 1500 mg radiolabelled norUDCA and a peak concentration reached a level [66] that is similar to those observed in the current mouse study. A more recent study evaluating pharmacokinetics of norUDCA showed maximum plasma concentration of ∼110 μM in healthy adults treated with 1500 mg/day [50]. Thus, norUDCA could impact systemic energy metabolism in humans, in particular under stress conditions. However, physiological differences in cardiac and bile acid metabolism between mice and humans may limit the study's conclusions. In contrast to rodents, norUDCA in humans is efficiently glucuronidated [62] and subsequently excreted via the urine, which may limit its plasma accumulation. Similarly, BAs in humans undergo amidation predominately with glycine and BA-sulfation is a unique characteristic in humans. Thus, it will be interesting to study preclinical models and patients undergoing surgical intervention with massive changes in bile acid pool compositions regarding its impact on cardiac and BAT fuel oxidation and energy expenditure and evaluate whether systemic levels of norUDCA may also comprise energy metabolism in humans, particular under stress conditions. Critically, our findings regarding the acute effects of norUDCA on murine peripheral metabolism should not be interpreted as evidence of direct clinical cardiotoxicity in humans. Instead of predicting adverse clinical events, these data underscore the distinct roles of unconjugated versus conjugated bile acids in modulating the metabolic sensitivity of the heart and BAT.

## 4 Material and methods

### 4.1 Animal models

All animal experiments were approved by the Behörde für Gesundheit und Verbraucherschutz Hamburg (Germany) or by the Austrian Federal Ministry of Education, Science and Research (Austria). All animals were housed with a 12-h light–dark cycle in humidity and temperature-controlled conditions and permitted ad libitum consumption of water and a standard mouse diet. *Cyp2c70*^*−/−*^, *ApoA5*^*−/−*^ and Atgl-MHC mice and the respective control littermates were bred and housed in the animal facility of the UKE or Graz. Mice were randomized based on body weight and fed for 7 days a chow diet (Altromin 1324) or a chow diet supplemented with norUDCA, UDCA, CDCA, DCA, CA, OCA (0.05%) or cholylsarcosin at 0.5% (w/w) with ad libitum access to food and water. For antibiotic treatment, neomycin (Sigma, #N6386), bacitracin (Sigma, #11702) and streptomycin (Sigma, #S6501) were diluted in the drinking water, all at a concentration of 1 g/L. Organ harvests and metabolic turnover studies were performed after a 4 h fasting period, and the mice were anesthetized with a lethal dose of ketamine and xylazine. Cardiac blood was drawn with syringes containing 0.5 M EDTA. Animals were perfused with PBS containing 10 U/ml heparin, and then the organs were harvested and immediately stored at −80 °C for further analysis. Body composition analysis was performed by echoMRI (Zinsser Analytic).

### 4.2 Indirect calorimetry

Indirect calorimetry was performed using either TSE Phenomaster system (TSE systems) or the PROMETHION systems (Sable Systems) in a temperature- and humidity-controlled chamber. During the experiments, all mice were housed in single cages under a 12 h light: 12 h dark cycle and had ad libitum access to food and water. The animals were fed regular chow with/without 0.5% norUDCA or a low-carb diet (ssniff, E15660) with/without 0.5% norUDCA.

### 4.3 Gene expression

Tissues were lysed in TRIzol (Ambion, Life Technologies) using a TissueLyser (Qiagen). Nucleic acids were extracted with chloroform, and total RNA was isolated using the RNA purification kit NucleoSpin®RNA II (Macherey & Nagel). RNA concentration was determined with NanoDrop, and 400 ng of RNA was used for reverse transcription into cDNA by using the III Reverse Transcriptase (Invitrogen). Quantitative real-time PCR was performed on a QuantStudio™ 5 Real-Time PCR System using the following TaqMan® on-demand primer sets (Invitrogen): *Acaca*: Mm01304285_m1, *Acly*: Mm00652520_m1, *Ccl2*: Mm00441242_m1, *Ccl5*: Mm01302428_m1, *Cd68*: Mm03047343_m1, *Col1a1*: Mm00801666_g1, *Cpt1*: Mm00550438_m1, *Cxcl10*: Mm00445235_m1, *Fasn*: Mm00662319_m1, *Il1b*:Mm00434228_m1, *Lipe*: Mm00495359_m1, *Lpl*: Mm00434764_m1, *Mmp12*: Mm00500554_m1, *Mmp13*: Mm00439491_m1), *Pdk4*: Mm00443325_m1, *Pfkfb4*: Mm00557176_m1, *Pnpla2*: Mm00503040_m1, *Ppargc1a*: Mm00447183_m1, *Scd1*: Mm00772290_m1, *Slc2a1* (encoding Glut1): Mm00441480_m1, *Slc2a4* (encoding Glut4): Mm01245502_m1, *Tbp*: Mm00446973_m1, *Timp1*: Mm00441818_m1, *Tnfa*: Mm00443258_m1). mRNA levels were normalized to the level of the housekeeping gene TATA-box binding protein (*Tbp*) mRNA, and the results were displayed as relative gene expression normalized to the experimental control group, following calculations using the 2-ΔΔCt method.

### 4.4 RNA sequencing

For transcriptomics, RNA was purified from total RNA using poly-T oligo-attached magnetic beads. After fragmentation, the first strand cDNA was synthesized utilizing random hexamer primers, followed by the second strand cDNA synthesis. Libraries prepared using Novogene NGS RNA Library Prep Set (PT042) was sequenced on Illumina NovaSeq 6000 platform S4 flow cell. RNA-seq data are aligned using Hisat2 v2.0.5. Reads are assigned to transcripts using featureCounts. Differential expression analysis of the data was performed using edgeR. The P values were adjusted using the Benjamini & Hochberg method. Local version of the gene set enrichment analysis tool http://www.broadinstitute.org/gsea/index.jsp was used, GO, KEGG (Kyoto Encyclopedia of genes and Genomens) and Reactome were used for GSEA independently.

### 4.5 Plasma parameters

Plasma was isolated by centrifugation of EDTA-spiked blood for 10 min at 10.000 rpm at 4 °C in a bench top centrifuge. Glucose was determined in tail blood using Accu-Check Aviva test strips (ROCHE). Non esterified fatty acids (NEFAs) were determined photometrically using the NEFA-HR (2) Assay (FUJIFILM). Ketone bodies were determined using the Autokit Total Ketone Bodies Assay (FUJIFILM). Glycerol was determined calorimetrically using the Free Glycerol Reagent (SIGMA, #F6428) and the Glycerol Standard Solution (SIGMA, #G7793) as reference.

### 4.6 Western blotting

For SDS–PAGE, perfused organs were harvested and homogenized with a TissueLyzer (Qiagen) in 10x excess of (v/w) RIPA buffer (50 mM Tris–HCl pH 7.4, 5 mM EDTA, 150 mM sodium chloride, 1 mM sodium pyrophosphate, 1 mM sodium fluoride, 1 mM sodium ortho-vanadate, 1% (NP-40) supplemented with cOmplete mini protease inhibitor cocktail tablets (Roche), and phosphatase inhibitor cocktail (Bimake.com). After centrifugation at 16.000*g* for 10 min, the clear soluble middle layer of the lysate was taken, and protein concentration was assessed using the method of Lowry. Then 20 μg of total protein was denatured at 55 °C for 10 min in a NuPAGE reducing sample buffer (Invitrogen) and separated on 10% SDS–polyacrylamide Tris–glycine gels. Proteins were transferred to nitrocellulose membranes in a wet blotting system. Equal loading was confirmed by Ponceau S (Serva) staining. Subsequently, the membranes were washed twice in TBS-T (20 mM Tris, 150 mM sodium chloride, 0.1% (v/v) Tween 20) and blocked for 1 h in 5% milk powder (Sigma) in TBS-T at room temperature. Primary antibodies were incubated (5% BSA in TBS-T) overnight at 4 °C, and secondary antibodies were diluted in 5% milk powder in TBS-T. Detection was performed with enhanced chemiluminescence using an Amersham Imager 600 (GE Healthcare). The following primary antibodies were used: rabbit polyclonal anti-CD36 (1:1000, Novus biologicals, NB400-144), goat polyclonal anti-LPL (1:1000, kind gift from Andre Bensadoun, Cornell University), rat monoclonal anti-GPIHBP1 (1:1000, kind gift from Stephen G. Young, UCLA), mouse monoclonal total OXPHOS WB cocktail (1:500, abcam, ab110413), rabbit monoclonal anti-gamma-Tubulin (1:1000, abcam, ab179503), rabbit monoclonal anti-AKT (1:1000, cell signaling, #9272). The following secondary antibodies (in a dilution of 1:5.000) were used: HRP goat anti-rabbit (Jackson ImmunoResearch Labs, #111-035-144), HRP goat anti-mouse (Jackson ImmunoResearch Labs, #115-035-003), HRP donkey anti-rat (Jackson ImmunoResearch Labs, #712-035-150), and HRP mouse anti-goat (Jackson ImmunoResearch Labs, #205-035-108).

### 4.7 Bile acid measurement

Bile acids were quantified by HPLC coupled to electrospray ionization tandem mass spectrometry as described [67]. Briefly, plasma or cecal samples were prepared by a methanol liquid–liquid extraction. Quantitative measurement of bile acids was performed using a LC-ESI-QqQ system run multiple reaction monitoring (MRM) mode. HPLC analysis was performed using NEXERA X2 LC-30AD HPLC PUMP (Shimadzu, Tokyo, Japan) equipped with a Kinetex C18 column (100 Å, 150 mm × 2.1 mm i.d., Phenomenex, Torrance, CA, USA). For HPLC a mobile phase A consisting of water and a mobile phase B consisting of acetonitrile methanol (3/1 v/v) both enriched with 0.1% formic acid and 20 mM ammonium acetate was used. The column was coupled to QqQ: Q trap 5500 System (SCIEX, Darmstadt, Germany). Peaks were identified and quantified by comparing retention times, as well as MRM transitions and peak areas, respectively, to particular corresponding standard chromatograms.

### 4.8 Metabolomic enrichment analysis

For flux analysis, mice treated with/without norUDCA received a bolus i.p. injection of uniformly labeled ^13^C-glucose (1 g/kg body weight). Mice were sacrified 30 min after injection and organs were harvested for metabolomics. Each x mg tissue sample was mixed with five times the μl amount of ice-cold extraction solvent acetonitrile (ACN) with H_2_O (1:1) and homogenized using a TissueLyser II (30 Hz, 10 min; Retsch Qiagen). After centrifugation (4 °C, 2 min, 14000 rpm), 250 μl supernatant were mixed with 250 μl ACN: H_2_O for a second extraction step. After vortexing for 1 min and centrifugation (4 °C, 10 min, 14000 rpm), the supernatant was transferred to a new tube and evaporated to dryness (SpeedVac, Eppendorf). Extracted metabolites of heart and BAT were analyzed by LC-MS/MS using an adapted method described by Buescher et al., 2010 [68]. Prior to measurement, each sample was re-suspended in 100 μl H_2_O and 10 μl were injected onto an Agilent 1290 II infinity UPLC system (Agilent Technologies Inc., Santa Clara, CA, USA) coupled on-line with a QTRAP® 6500+ mass spectrometer (Sciex, Framingham, USA). Chromatographic separation was achieved with a XSelect HSS T3 XP column (2.1 × 150 mm, 2.5 μm, 100 Å; Waters, Milford, MA, USA) connected to an XP VanGuard® cartridge (HSS T3, 2.1 × 5 mm, 2.5 μM; Waters, Milford, MA, USA). Mobile phase A and mobile phase B were 10 mM tributylamine, 10 mM acetic acid, 5% methanol and 2% 2-propanol (pH 7.1) in water and 100% 2-propanol, respectively. Metabolites were eluted with the following non-linear gradient: 0–15.5 min 0.4 mL/min, 15.5–16.5 min 0.4–0.15 mL/min, 16.5–23 min 0.15 mL/min, 23–27 min 0.15–0.4 mL/min, 27–33 min 0.4 mL/min. The autosampler was kept at 5 °C and the column oven was set to 40 °C. For identification and quantitation, a scheduled multiple reaction monitoring (MRM) method in negative mode electrospray ionization was used with specific transitions for every metabolite and isotopologue. Data acquisition was performed using the Analyst® software (v. 1.7.0) and peak integration was done in SciexOS® Software (v. 3.0.0., Sciex). All isotopologue measurement values were corrected for 1.1% of ^13^C-natural abundance [69].

### 4.9 Metabolic turnover studies

To quantify the uptake of radiolabeled bile acids, mice received an oral gavage enriched with ^3^H-norUDCA (74 kBq per mouse) and ^14^C-CA (37 kBq per mouse). Four hours after gavage, mice were anaesthetized and organs were harvested. To quantify the uptake of energy substrates, mice were i.v. injected with ^14^C-DOG (7.4 kBq per mouse), ^3^H-DOG (14.8 kBq per mouse), albumin-bound ^14^C-oleic acid (7.4 kBq per mouse) or ^14^C-hydroxybutyrate (7.4 kBq per mouse). Mice were anaesthetized 15 min after injection, blood was collected by cardiac puncture, perfused with PBS via the left heart ventricle and organs were harvested. In both setups, organs were dissolved in Solvable (Perkin Elmer) for scintillation counting using a Perkin Elmer Tricarb Scintillation Counter.

### 4.10 Histology and immunohistochemistry

Immunohistochemical stainings were performed on paraffin-embedded tissues using standard procedures. Briefly, liver tissues were fixed in 3.7% formaldehyde in PBS solution and later embedded in paraffin. Stainings were performed using 4 μm sections cut on a Leica microtome and mounted on Histobond slides (Marienfeld-Superior). The following primary antibodies were used in 3% BSA (Sigma): rat monoclonal anti-LY6C (1:200, abcam, ab15627), rabbit monoclonal anti-CK19 (1:200, abcam, ab52625). Horseradish peroxidase (HRP) coupled donkey-anti-rat (Jackson Immunoresearch, #712-036-153) and horseradish peroxidase (HRP) goat anti-rabbit (Jackson ImmunoResearch Labs, #111-035-144) were used as secondary antibodies. After secondary antibody incubation, sections were washed with PBS 3-times for 10 min. Staining was performed using an abcam DAB kit following the manufacturer's instructions. After DAB-staining, the slides were rinsed with PBS to stop the chromogenic reaction and counterstained with hematoxilin for 2 min. Slides were incubated under running tap water for 10 min to achieve bluing of the hematoxilin. Afterwards, the slides were dehydrated and mounted using Eukitt. Images were taken using a NikonA1 Ti microscope equipped with a DS-Fi-U3 brightfield camera.

### 4.11 Ex vivo lipolysis assay

White adipose tissue pieces (∼25–35 mg) were incubated in 500 μl Dulbecco's Modified Eagle Medium (Gibco, #11965092) supplemented with 2% fatty acid-free bovine serum albumin (Sigma, #A8806). After 30 min, basal lipolysis was determined by measuring the released free fatty acids and glycerol in the media, using the NEFA-HR (2) Assay (FUJIFILM) and the Free Glycerol Reagent (SIGMA, #F6428), respectively. Protein content of the individual adipose tissue explant pieces was measured with the Lowry method for normalization.

### 4.12 Engineered heart tissues

An established control line of human induced pluripotent stem cells (hiPSC, hiPSCreg code: UKEi001-A, ERC001 XX) was used to differentiate cardiomyocytes as recently described [70]. Briefly, master/working cell bank hiPSCs aliquots [71] were expanded in FTDA media on Geltrex-coated cell culture vessels [72]. Embryoid bodies were generated in spinner flasks. Ventricular cardiomyocytes were differentiated in suspension/EB format by growth factor/small molecule cocktails into mesodermal progenitor cells and subsequently into cardiomyocytes. Collagenase-dissociated hiPSC-CM were either cryopreserved or used directly for the generation of engineered heart tissues (EHT). Differentiation efficiency was determined by FACS analysis for troponin T. Fibrin-based strip-format EHTs were generated with 1.0 × 106 hiPSC-CM per construct [70]. EHTs were cultivated for approximately 21 days in EHT medium (10% horse serum, 1% penicillin-streptomycin, 33 μg/mL aprotinin, 10 μg/mL insulin, 200 μM tranexamic acid), in 24 well plates. Cell culture media was changed on Mondays, Wednesday and Fridays. Functional assessment was performed by video-optical recording of spontaneous EHT contraction and calculation of force based on deflection during contraction [73]. EHT were equilibrated in measurement medium (DMEM, horse serum 2%) overnight. After baseline contractility recording EHTs were incubated in the presence of vehicle (0.9% NaCl) or bile acids. Recording of contractility was performed at 2 h, 24 h, 48 h, and 72 h of incubation.

### 4.13 Electron microscopy

For electron microscopy mice were sacrificed with a lethal dose of ketamin/xylazine injection anesthesia and perfused with PBS. Organs were cut and directly transferred into fixative (4% PFA, 1% GA in PBS) and stored at 4 °C. Then, tissues were dissected with a razor blade and rinsed three times in 0.1 M sodium cacodylate buffer (pH 7.2–7.4) and osmicated using 1% osmium tetroxide in cacodylate buffer. Following osmication, the samples were dehydrated using ascending ethyl alcohol concentration steps, followed by two rinses in propylene oxide. Infiltration of the embedding medium was performed by immersing the pieces in a 1:1 mixture of propylene oxide and Epon and finally in neat Epon and hardened at 60 °C. Semithin sections (0.5 μm) were prepared for light microscopy mounted on glass slides and stained for 1 min with 1% Toluidine blue. Ultrathin sections (60 nm) were cut and mounted on copper grids. Sections were stained using uranyl acetate and lead citrate. Thin sections were examined and photographed using an EM902 (Zeiss) electron microscope.

### 4.14 Statistical analyses

Data are expressed as mean ± S.E.M. Comparisons of two groups were examined using Students T-Test. Comparison of three or more groups were analyzed using ANOVA. GraphPad Prism and Microsoft Excel were used for all statistical analyses. The statistical parameters can be found in the figure legends. P < 0.05 was considered to be statistically significant.

## CRediT authorship contribution statement

**Ioannis Evangelakos:** Writing – review & editing, Investigation, Formal analysis, Data curation, Conceptualization. **Esther Verkade:** Writing – review & editing, Methodology, Investigation. **Julia K. Rohde:** Writing – review & editing, Methodology, Investigation. **Alex Zaufel:** Writing – review & editing, Resources, Methodology, Investigation, Funding acquisition. **Martin Vargek:** Writing – review & editing, Methodology, Investigation, Funding acquisition, Conceptualization. **Markus Heine:** Writing – review & editing, Methodology, Investigation. **Anna Worthmann:** Writing – review & editing, Methodology, Investigation, Funding acquisition, Data curation. **Sebastian Graute:** Writing – review & editing, Methodology, Investigation. **Marceline Manka Fuh:** Writing – review & editing, Methodology, Investigation, Data curation. **Karthikeyan Gunasekaran:** Writing – review & editing, Methodology, Investigation. **Manju Kumari:** Writing – review & editing, Methodology, Investigation. **Dorothee Schwinge:** Writing – review & editing, Methodology, Investigation. **Martin von Bergen:** Writing – review & editing, Resources, Investigation, Conceptualization. **Ulrike Rolle-Kampczyk:** Writing – review & editing, Methodology, Investigation, Formal analysis. **Beatrice Engelmann:** Writing – review & editing, Methodology, Investigation. **Rolf Breinbauer:** Writing – review & editing, Resources, Methodology, Investigation, Formal analysis. **Rita Fuerst:** Writing – review & editing, Resources, Methodology, Investigation. **Umber Saleem:** Writing – review & editing, Methodology, Investigation. **Jan Freark de Boer:** Writing – review & editing, Resources, Investigation. **Christian Schlein:** Writing – review & editing, Methodology, Investigation, Funding acquisition. **Arne Hansen:** Writing – review & editing, Writing – original draft, Resources, Methodology, Investigation, Conceptualization. **Ludger Scheja:** Writing – review & editing, Writing – original draft, Methodology, Funding acquisition, Conceptualization. **Folkert Kuipers:** Writing – review & editing, Writing – original draft, Supervision, Funding acquisition, Conceptualization. **Tarek Moustafa:** Writing – review & editing, Writing – original draft, Supervision, Methodology, Investigation, Funding acquisition, Conceptualization. **Joerg Heeren:** Writing – review & editing, Writing – original draft, Supervision, Investigation, Funding acquisition, Conceptualization.

## Declaration of competing interest

The authors declare that they have no known competing financial interests or personal relationships that could have appeared to influence the work reported in this paper.

## Data Availability

Data will be made available on request.
