# Supplementary material for: The conjugation-resistant bile acid norUDCA cures liver fibrosis but impairs systemic energy metabolism
Source: Mol Metab. 2026 Apr 2;107:102363. doi: 10.1016/j.molmet.2026.102363 (PMC13096911; doi:10.1016/j.molmet.2026.102363)
Supplement: Multimedia component 1 [file mmc1.pdf]

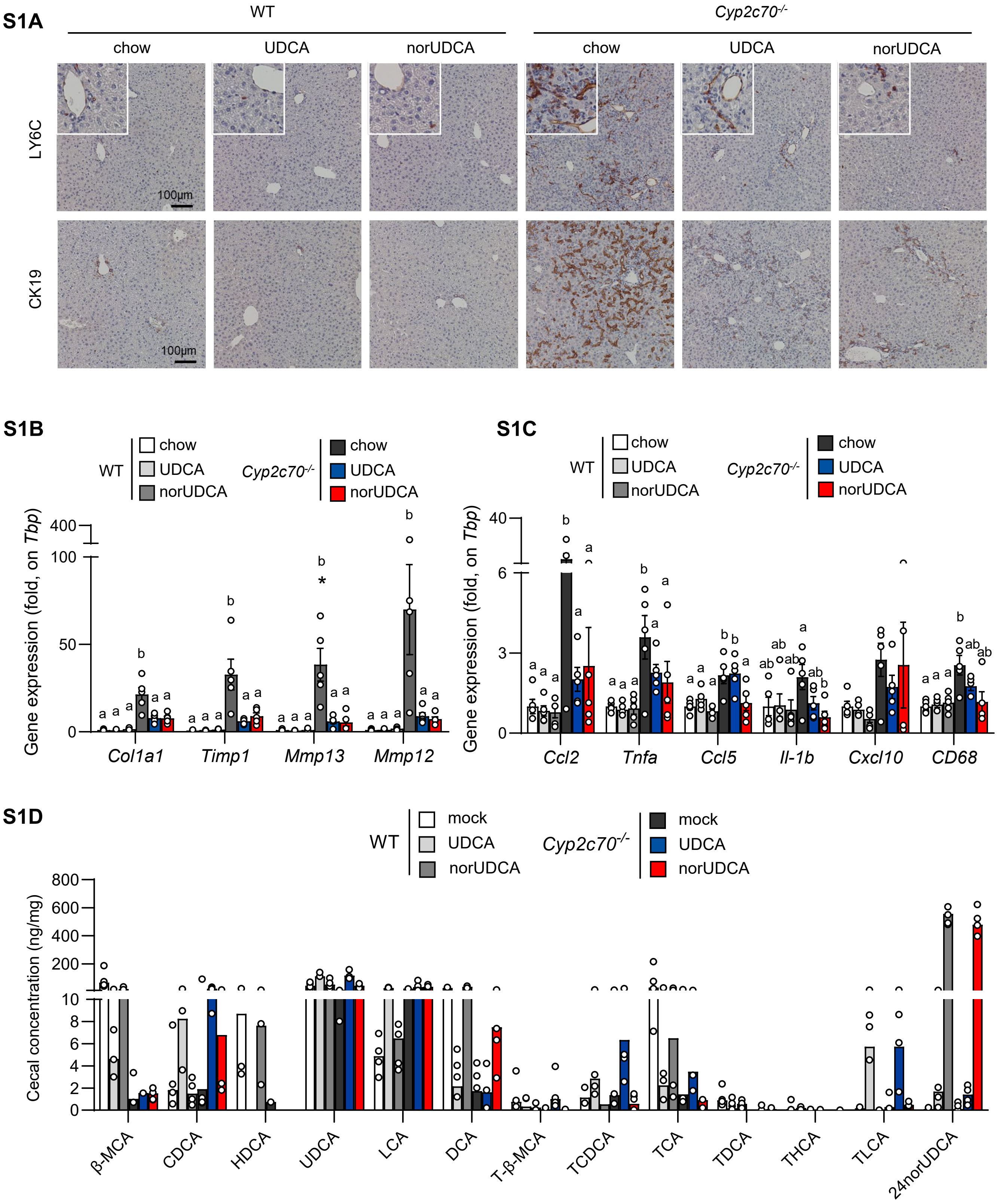

Figure S1. NorUDCA and UDCA alleviate liver inflammation and fibrosis in *Cyp2c70$^{-/-}$* mice. Wild type and *Cyp2c70$^{-/-}$* mice were fed a regular chow, or chow diet supplemented with either 0.5% UDCA or 0.5% norUDCA for 7 days under standard housing conditions (n=4-5). **A** Representative images of LY6C and CK19 stainings of liver sections. b-d, Hepatic expression of fibrosis marker genes (**B**), inflammation marker genes (**C**), and cecal bile acid concentration (**D**). Error bars are shown as SEM. Statistical analysis was performed two-way ANOVA (same letter denotes groups that are not significantly different from each other, with $p < 0.05$).

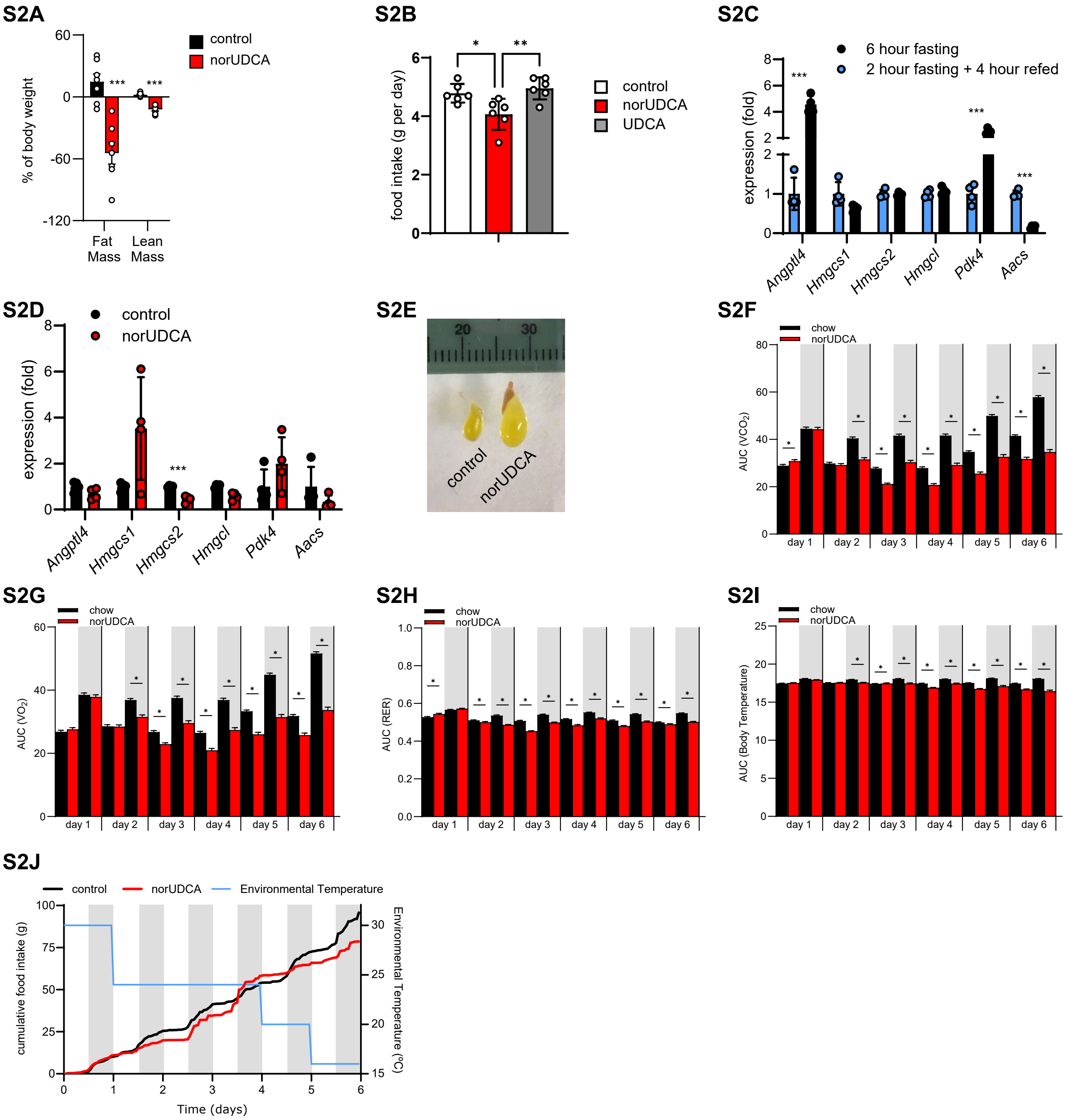

Figure S2. NorUDCA reduces fat mass and increases gall bladder size. Mice were fed a regular chow (control), or chow diet supplemented with norUDCA for 7 days with housing at room temperature. **A** Change of fat and lean mass determined by echoMRI (*n*=7). **B** Mean food intake per day determined between day 3 and day 7 of treatment. **C-D** Hepatic gene expression determined by RNAseq of mice, which were fasted for 6 hours or fasted for 2 hours and refed for 4 hours (**C**) or treated with control or norUDCA diet (**D**). **E** Representative image of gallbladder size of chow (control) and norUDCA-treated mouse under standard housing conditions. **F-J** Quantification of indirect calorimetry, body temperature and food intake measurements from the experiments shown in Fig. 1 separately calculated for the light and dark phases. CO_2 production (**F**), O_2 consumption (**G**), respiratory exchange rate (**H**), body core temperature (**I**). **J** Cumulative food intake from the experiments shown in Fig. 1. Error bars indicate standard error of the mean (SEM). Statistical analysis was performed with Student's T-Test. *p<0.05, **p<0.01,***p<0.005.

Extended Data Figure 3

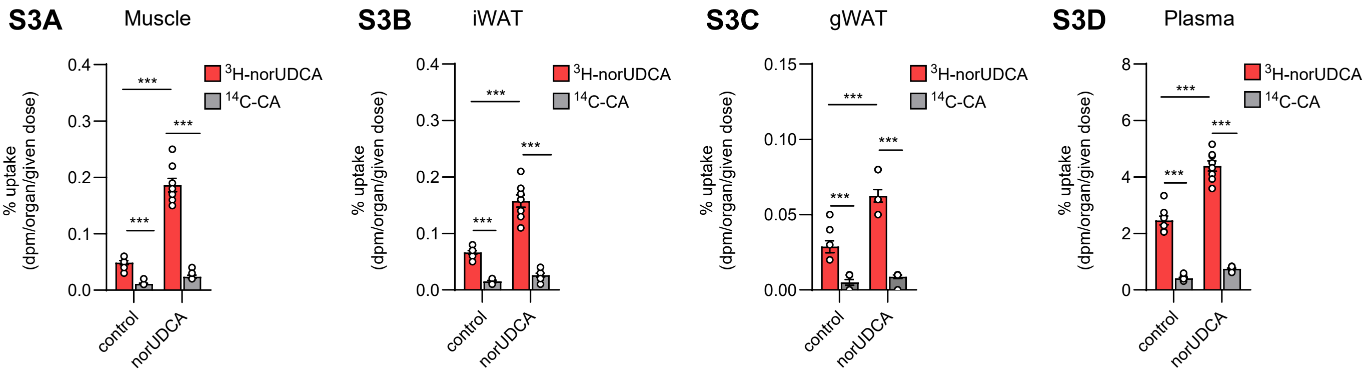

Figure S3. norUDCA accumulates in metabolically active organs. A-D Wild type mice were fed a regular chow (control), or chow diet supplemented with 0.5% norUDCA before oral administration of ^{3}H-norUDCA and ^{14}C-CA (*n=8*). Four hours later, percentage of applied tracers was determined in muscle (**A**), inguinal white adipose tissue (iWAT) (**B**), gonadal white adipose tissue (gWAT) (**C**), and plasma (**D**). Statistical analysis was performed with two-way ANOVA (**A-D**). *p<0.05, **p<0.01,***p<0.005.

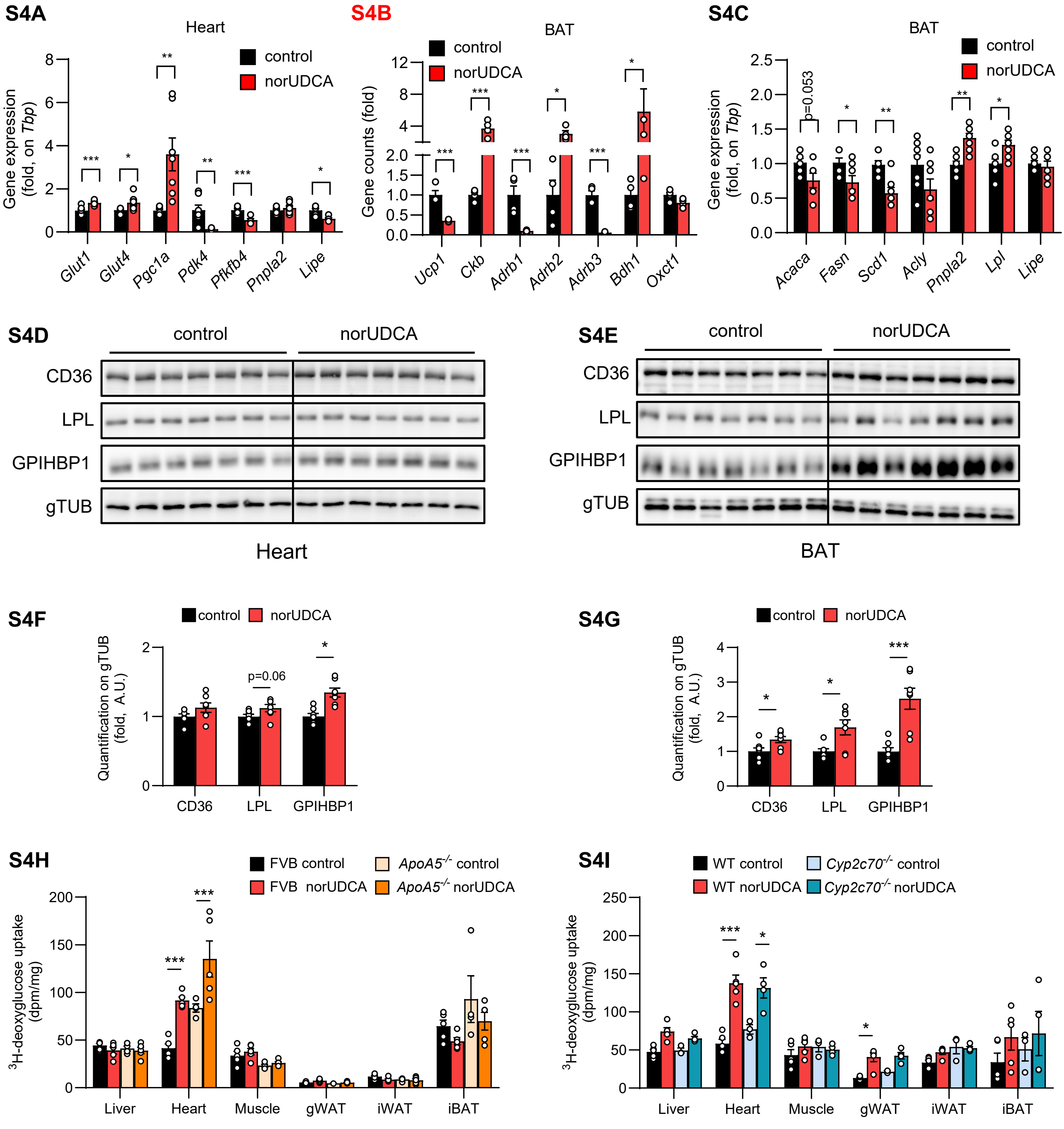

Figure S4. norUDCA shifts cardiac energy metabolism towards glucose utilization. Mice were fed a regular chow (control), or chow diet supplemented with norUDCA at 0.5% for 7 days with housing at room temperature. **A** Expression of metabolic genes in heart measured by qPCR. **B** Expression of genes determining thermogenesis, adrenergic signaling and ketone body metabolism in BAT. Fold expression was calculated from RNAseq data, **C** Expression of metabolic genes in BAT measured by qPCR. **D-G**, Western blot analysis and quantification of lipoprotein-processing proteins from samples of heart (**D, F**) and BAT (**E, G**). **H-I**, Organ-specific uptake of intravenously administered ^{3}H-deoxyglucose in FVB wild type vs *ApoA5*$^{-/-}$ mice on the FVB background (**H**), and in wild type controls vs *Cyp2c70*$^{-/-}$ mice (**I**). Error bars indicate standard error of the mean (SEM). Statistical analysis was performed either with Student's T-Test (**A-G**) or two-way ANOVA (**H-I**). *= p<0.05, **= p<0.01,***= p<0.005.

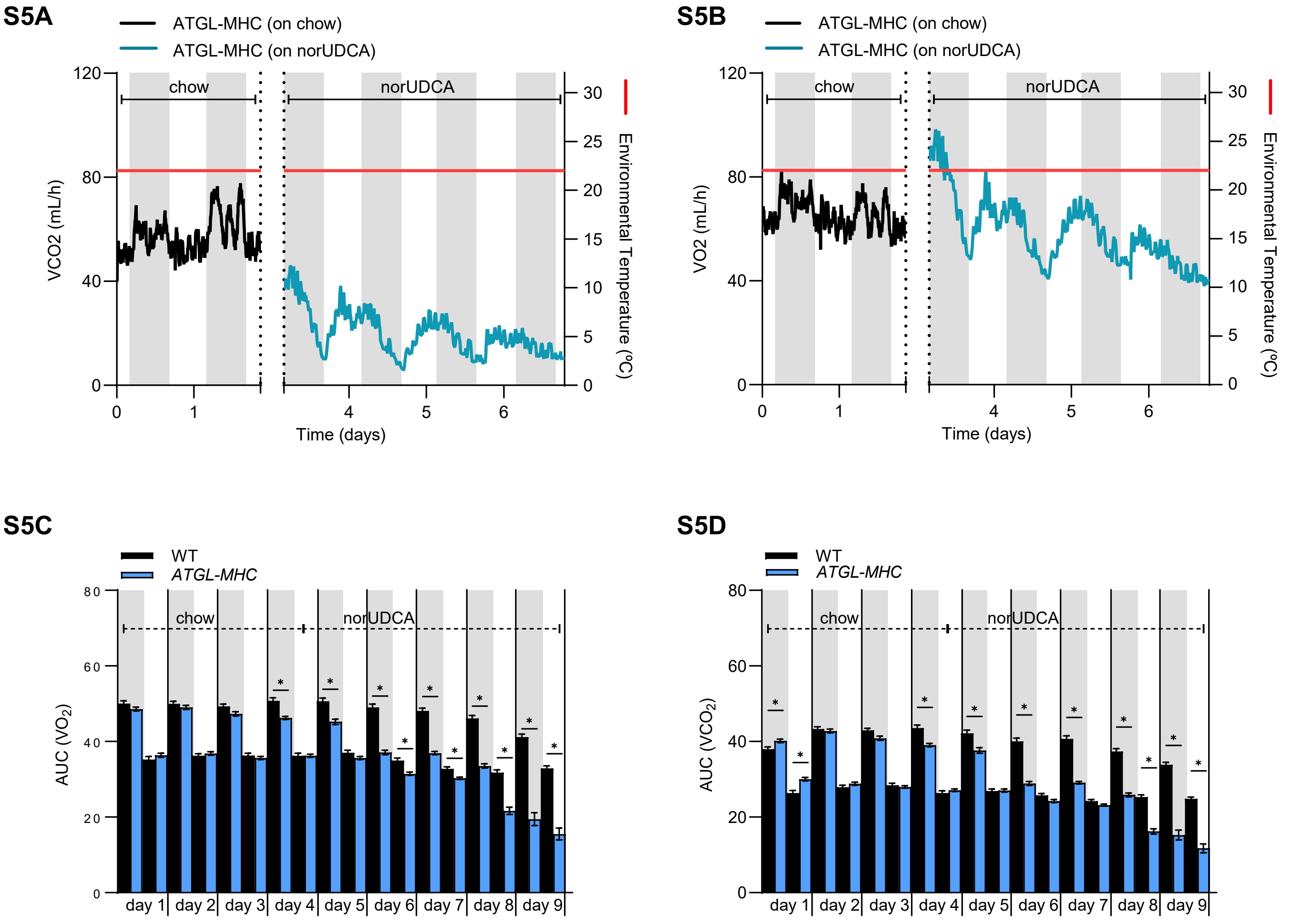

Figure S5. ATGL compensates for norUDCA-induced defects in energy homeostasis and thermogenesis at room temperature. A-D Indirect calorimetry of ATGL-MHC mice fed with chow followed by norUDCA-enriched diet for the indicated time period at room temperature. CO_2 production (**A**), O_2 consumption (**B**), quantification of indirect calorimetry measurements separately calculated for the light and dark phases for CO_2 production (**C**) and O_2 consumption (**D**). Error bars indicate standard error of the mean (SEM). Statistical analysis was performed with Student's T-Test. *= $p<0.05$.

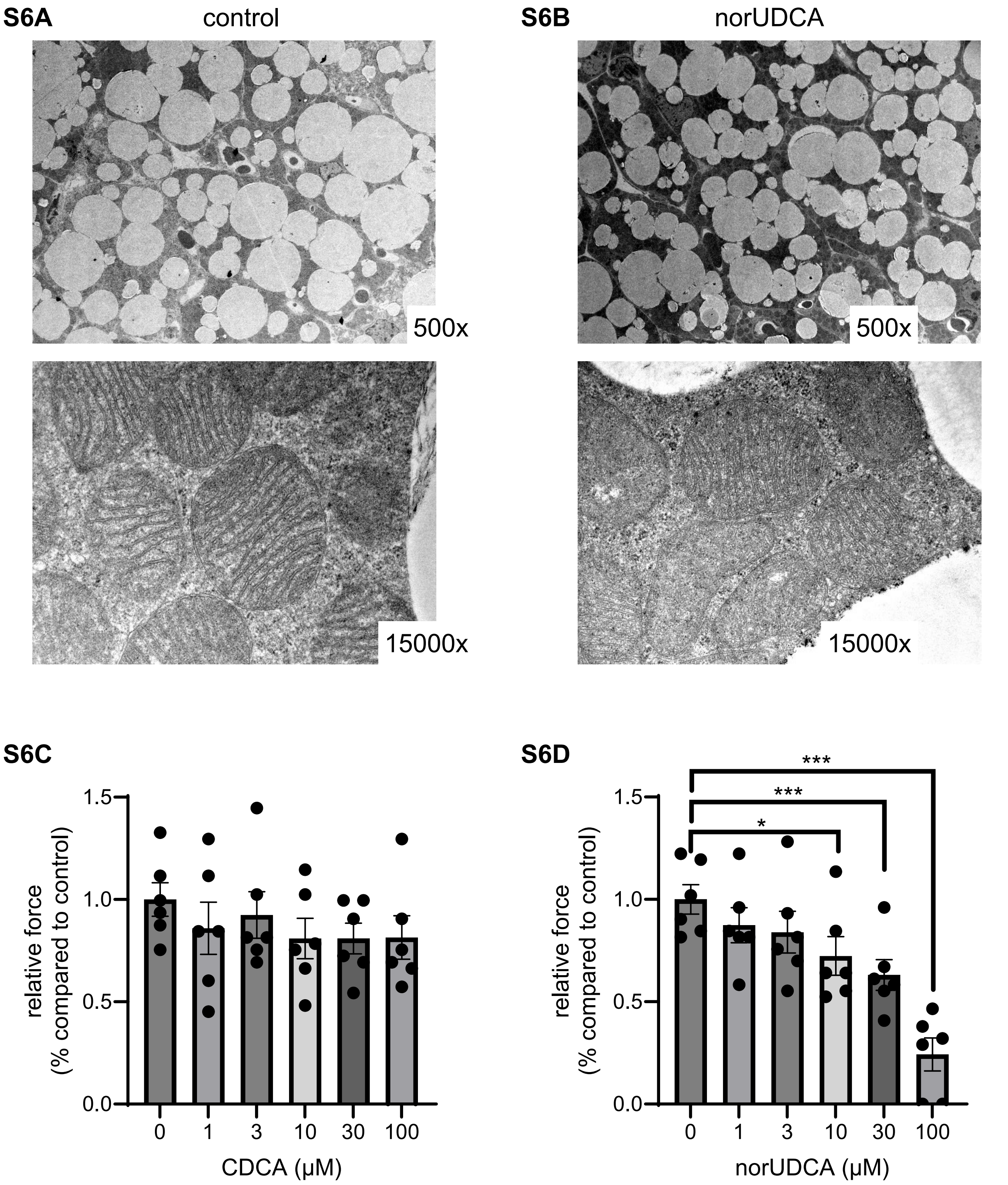

Figure S6. Electron microscopy of BAT from mice and model depicting the effects of norUDCA on metabolically active organs at room temperature and at cold. A-B, Mice were fed a regular chow (control), or chow diet supplemented with norUDCA for 7 days with housing at room temperature. Representative electron microscopy pictures of BAT from control (**A**) and norUDCA-treated mice (**B**). **C-D** Engineered heart tissues (EHTs) were generated from human induced pluripotent stem cells (hiPSCs) and incubated with indicated concentrations of CDCA (**C**) or norUDCA (**D**). The relative force were measured (n=6). Error bars are shown as SEM. Statistical analysis was performed by Student's T-Test.
