# Supplementary material for: The conjugation-resistant bile acid norUDCA cures liver fibrosis but impairs systemic energy metabolism
Source: Mol Metab. 2026 Apr 2;107:102363. doi: 10.1016/j.molmet.2026.102363 (PMC13096911; doi:10.1016/j.molmet.2026.102363)
Supplement: Multimedia component 2 [file mmc2.pdf]

gene_id	Control	norUDCA	log2FoldChange	padj	gene_name
ENSMUSG00000032942	3621	162	-4.476	8.6E-136	Ucp3
ENSMUSG00000020241	2347	642	-1.871	1.7E-113	Col6a2
ENSMUSG00000034958	176	1456	3.051	1.3E-98	Atcay
ENSMUSG00000085779	1410	9471	2.749	1.5E-92	Atcayos
ENSMUSG00000044338	773	36	-4.438	1.7E-90	Aplnr
ENSMUSG00000023034	343	7726	4.494	1.3E-88	Nr4a1
ENSMUSG00000026043	7127	487	-3.871	4.4E-86	Col3a1
ENSMUSG00000019577	45698	7525	-2.602	4.4E-86	Pdk4
ENSMUSG00000038894	595	12908	4.439	3.8E-84	Irs2
ENSMUSG00000028339	2676	369	-2.862	7.5E-83	Col15a1
ENSMUSG00000028150	1450	5583	1.946	2.6E-72	Rorc
ENSMUSG00000027956	1540	7508	2.285	1.4E-68	Tmem144
ENSMUSG00000031530	116	2824	4.600	2.5E-67	Dusp4
ENSMUSG00000038546	1456	4835	1.731	2.0E-65	Ranbp9
ENSMUSG00000107428	9	242	4.703	6.2E-63	Gm44154
ENSMUSG00000053093	17707	291868	4.043	5.9E-61	Myh7
ENSMUSG00000001506	2813	595	-2.242	1.3E-60	Col1a1
ENSMUSG00000029661	4017	1176	-1.771	1.4E-57	Col1a2
ENSMUSG00000112831	131	1385	3.395	3.0E-57	AC167229.1
ENSMUSG00000022526	649	1691	1.386	7.0E-57	Zfp251
ENSMUSG00000045193	2135	5804	1.442	1.2E-54	Cirbp
ENSMUSG00000086645	44	461	3.393	1.9E-54	Gm15743
ENSMUSG00000028607	6875	2620	-1.392	2.2E-53	Cpt2
ENSMUSG00000026414	301776	601871	0.996	3.1E-51	Tnnt2
ENSMUSG00000061175	1139	6846	2.587	1.1E-49	Fnip2
ENSMUSG00000001627	1203	5684	2.240	2.8E-49	Ifrd1
ENSMUSG00000032177	2321	916	-1.341	3.5E-49	Pde4a
ENSMUSG00000070436	6560	2066	-1.667	4.4E-49	Serpinh1
ENSMUSG00000022270	12095	50168	2.052	6.5E-49	Retreg1
ENSMUSG00000013089	723	2077	1.522	1.1E-48	Etv5
ENSMUSG00000003849	1737	4352	1.324	1.1E-48	Nqo1
ENSMUSG00000037519	2306	7037	1.609	6.8E-48	Ppfia1
ENSMUSG00000111815	184	1003	2.452	9.8E-47	Gm6018
ENSMUSG00000015852	265	18	-3.905	9.8E-47	Fcrls
ENSMUSG00000024011	2504	324	-2.952	1.2E-45	Pi16
ENSMUSG00000049999	524	110	-2.247	3.0E-45	Ppp1r3d
ENSMUSG00000029135	1179	5825	2.305	1.9E-43	Fosl2
ENSMUSG00000046916	663	195	-1.765	2.2E-43	Myct1
ENSMUSG00000087221	178	596	1.748	1.1E-42	BC037032
ENSMUSG00000002289	785	69	-3.497	1.7E-42	Angptl4
ENSMUSG00000026628	122	1597	3.702	4.7E-42	Atf3
ENSMUSG00000037010	512	135	-1.927	7.4E-42	Apln
ENSMUSG00000027238	5009	25031	2.321	1.8E-41	Frmd5
ENSMUSG00000028834	8910	42605	2.257	4.4E-41	Trim63
ENSMUSG00000040950	374	71	-2.402	4.8E-41	Mgl2
ENSMUSG00000031461	57875	136216	1.235	2.1E-40	Myom2
ENSMUSG00000042834	513	88	-2.541	4.1E-40	Nrep
ENSMUSG00000029167	4935	31202	2.661	1.1E-39	Ppargc1a
ENSMUSG00000044991	1359	4917	1.856	3.9E-39	Shld1
ENSMUSG00000033849	286	30	-3.284	1.1E-38	B3galt2
ENSMUSG00000025612	1695	4660	1.459	1.6E-38	Bach1

ENSMUSG00000048126	1264	245	-2.372	4.2E-38	Col6a3
ENSMUSG00000031490	2461	5204	1.079	9.2E-38	Eif4ebp1
ENSMUSG00000024892	1572	2713	0.788	3.1E-37	Pcx
ENSMUSG00000026360	1560	687	-1.183	9.7E-37	Rgs2
ENSMUSG00000090015	139	413	1.575	1.3E-36	Gm15446
ENSMUSG00000020674	2346	805	-1.544	9.3E-36	Pxdn
ENSMUSG00000056481	283	64	-2.130	1.7E-35	Cd248
ENSMUSG00000024042	1010	7200	2.833	1.1E-34	Sik1
ENSMUSG00000015090	17080	68470	2.003	1.2E-34	Ptgds
ENSMUSG00000095438	126	497	1.992	2.4E-34	Mir133a-1hg
ENSMUSG00000102856	21	274	3.729	2.5E-34	Gm37084
ENSMUSG00000016494	3537	1489	-1.249	4.1E-34	Cd34
ENSMUSG00000024661	40577	67442	0.733	1.7E-33	Fth1
ENSMUSG00000050296	299	820	1.455	4.1E-33	Abca12
ENSMUSG00000079105	465	115	-2.009	6.3E-33	C7
ENSMUSG00000069662	1549	471	-1.717	6.3E-33	Marcks
ENSMUSG00000045094	245	990	2.009	9.4E-33	Arhgef37
ENSMUSG00000001119	2676	896	-1.578	2.2E-32	Col6a1
ENSMUSG00000038403	6912	3776	-0.872	2.6E-32	Hfe2
ENSMUSG00000017754	650	204	-1.679	3.0E-32	Pltp
ENSMUSG00000030643	232	777	1.746	4.5E-32	Rab30
ENSMUSG00000047617	501	1458	1.541	7.3E-32	Paxx
ENSMUSG00000036256	4821	1951	-1.305	1.1E-31	Igfbp7
ENSMUSG00000028530	6037	11187	0.890	1.1E-31	Jak1
ENSMUSG00000032411	5055	10346	1.033	1.2E-31	Tfdp2
ENSMUSG00000036206	665	1908	1.518	1.9E-31	Sh3bp4
ENSMUSG00000069495	854	1711	1.003	4.0E-31	Epc2
ENSMUSG00000085162	218	569	1.382	4.2E-31	Gm12295
ENSMUSG00000018593	10873	3606	-1.592	7.6E-31	Sparc
ENSMUSG00000020937	627	1980	1.660	5.1E-30	Plcd3
ENSMUSG00000036944	351	807	1.198	1.5E-29	Tmem71
ENSMUSG00000015837	14436	36222	1.327	1.9E-29	Sqstm1
ENSMUSG00000027397	701	2047	1.545	2.2E-29	Slc20a1
ENSMUSG00000005958	323	884	1.449	2.8E-29	Ephb3
ENSMUSG00000030967	2066	4390	1.087	3.6E-29	Zranb1
ENSMUSG00000097729	761	1697	1.159	8.3E-29	2310015A10Rik
ENSMUSG00000105541	37	195	2.369	9.9E-29	Gm43136
ENSMUSG00000109005	8	158	4.259	1.1E-28	Gm45221
ENSMUSG00000020902	3991	8942	1.164	1.1E-28	Ntn1
ENSMUSG00000043953	505	2544	2.332	1.2E-28	Ccrl2
ENSMUSG00000035283	730	159	-2.192	1.4E-28	Adrb1
ENSMUSG00000036181	3767	13465	1.838	1.5E-28	Hist1h1c
ENSMUSG00000028211	760	2535	1.737	1.6E-28	Trp53inp1
ENSMUSG00000053716	1892	676	-1.488	2.0E-28	Dusp7
ENSMUSG00000020300	2594	8012	1.627	2.2E-28	Cpeb4
ENSMUSG00000021477	6640	20106	1.598	3.5E-28	Ctsl
ENSMUSG00000041361	20494	35992	0.812	3.6E-28	Myzap
ENSMUSG00000100954	172	470	1.452	4.0E-28	Gm10138
ENSMUSG00000055632	1973	445	-2.150	6.0E-28	Hmcn2
ENSMUSG00000090291	15	130	3.154	1.2E-27	Lrrc10b
ENSMUSG00000086247	91	384	2.067	2.3E-27	Gm15787
ENSMUSG00000032350	545	1363	1.320	2.5E-27	Gclc

ENSMUSG00000016528	12263	26272	1.099	5.9E-27	Mapkapk2
ENSMUSG00000046480	1517	584	-1.378	6.5E-27	Scn4b
ENSMUSG00000021226	3096	1314	-1.236	2.1E-26	Acot2
ENSMUSG00000045045	393	137	-1.522	2.8E-26	Lrfn4
ENSMUSG00000051335	1946	5977	1.619	3.1E-26	Gfod1
ENSMUSG00000020542	1449	3665	1.338	4.1E-26	Myocd
ENSMUSG00000026728	7082	3177	-1.157	5.7E-26	Vim
ENSMUSG00000040841	477	195	-1.292	5.7E-26	Six5
ENSMUSG00000028271	534	2066	1.953	6.4E-26	Gtf2b
ENSMUSG00000031636	594	2507	2.079	9.5E-26	Pdlim3
ENSMUSG00000086430	12	170	3.854	1.3E-25	4930551O13Rik
ENSMUSG00000078349	247	708	1.523	2.8E-25	AW011738
ENSMUSG00000038463	349	1179	1.759	3.0E-25	Olfml2b
ENSMUSG00000042406	2182	4647	1.090	3.7E-25	Atf4
ENSMUSG00000041930	50	487	3.274	5.5E-25	Fam222a
ENSMUSG00000029314	442	167	-1.409	9.3E-25	Gpat3
ENSMUSG00000031659	603	223	-1.436	1.1E-24	Adcy7
ENSMUSG00000020178	365	77	-2.245	1.3E-24	Adora2a
ENSMUSG00000025790	4159	8786	1.078	2.1E-24	Slco3a1
ENSMUSG00000086290	169	467	1.463	2.6E-24	Snhg12
ENSMUSG00000038594	884	1853	1.065	3.9E-24	Cep85l
ENSMUSG00000005514	1778	3083	0.793	4.5E-24	Por
ENSMUSG00000074264	406	876	1.110	4.7E-24	Amy1
ENSMUSG00000006418	3012	6712	1.156	5.7E-24	Rnf114
ENSMUSG00000028341	174	3104	4.156	7.5E-24	Nr4a3
ENSMUSG00000039157	549	147	-1.905	8.5E-24	Fam102a
ENSMUSG00000022237	1462	4035	1.465	1.0E-23	Ankrd33b
ENSMUSG00000056260	526	1034	0.976	1.1E-23	Lrif1
ENSMUSG00000064145	1946	3479	0.837	1.5E-23	Arih2
ENSMUSG00000069763	734	1707	1.217	1.8E-23	Tmem100
ENSMUSG00000014470	1578	2865	0.859	1.9E-23	Rnf166
ENSMUSG00000008348	1962	4892	1.318	1.9E-23	Ubc
ENSMUSG00000021223	576	98	-2.562	2.0E-23	Papln
ENSMUSG00000015202	1350	4203	1.638	2.1E-23	Cnksr3
ENSMUSG00000071226	737	329	-1.162	2.5E-23	Cecr2
ENSMUSG00000050989	827	400	-1.052	3.2E-23	Selenon
ENSMUSG00000026672	4381	10056	1.199	4.7E-23	Optn
ENSMUSG00000025453	75	317	2.085	6.1E-23	Nnt
ENSMUSG00000097675	29	148	2.354	1.2E-22	1700101I11Rik
ENSMUSG00000027750	1321	282	-2.224	1.3E-22	Postn
ENSMUSG00000050556	2341	1066	-1.136	1.4E-22	Kcnb1
ENSMUSG00000031972	6911	1415	-2.288	1.4E-22	Acta1
ENSMUSG00000036533	810	383	-1.078	1.5E-22	Cdc42ep3
ENSMUSG00000031666	1804	2926	0.698	1.5E-22	Rbl2
ENSMUSG00000035279	270	62	-2.115	1.6E-22	Ssc5d
ENSMUSG00000102095	19	113	2.573	1.9E-22	C730036E19Rik
ENSMUSG00000022661	1555	558	-1.483	2.0E-22	Cd200
ENSMUSG00000024905	164	34	-2.272	2.3E-22	Tesmin
ENSMUSG00000030161	4308	11969	1.474	2.6E-22	Gabarapl1
ENSMUSG00000037185	119	347	1.531	3.2E-22	Krt80
ENSMUSG00000036492	45	215	2.245	3.6E-22	Rnf39
ENSMUSG00000056749	317	1864	2.555	5.1E-22	Nfil3

ENSMUSG00000060187	5788	13936	1.268	5.1E-22	Lrrc10
ENSMUSG00000001751	421	817	0.955	5.2E-22	Naglu
ENSMUSG00000032363	1319	408	-1.690	6.4E-22	Adamts7
ENSMUSG00000049985	21	122	2.574	7.6E-22	Ankrd55
ENSMUSG00000026885	118	423	1.842	8.7E-22	Ttll11
ENSMUSG00000031167	1803	4577	1.344	1.1E-21	Rbm3
ENSMUSG00000030560	1575	753	-1.065	1.1E-21	Ctsc
ENSMUSG00000096146	5268	7982	0.600	1.2E-21	Kcnj11
ENSMUSG00000050627	2886	4626	0.681	1.5E-21	Gpd1l
ENSMUSG00000097462	141	341	1.288	1.7E-21	9530026P05Rik
ENSMUSG00000009633	868	278	-1.641	2.3E-21	G0s2
ENSMUSG00000031485	2776	4904	0.820	2.6E-21	Plpbp
ENSMUSG00000026971	238	39	-2.614	2.6E-21	Itgb6
ENSMUSG00000117310	49	228	2.236	3.0E-21	Ptp4a1
ENSMUSG00000034442	1222	3067	1.329	3.6E-21	Trmt5
ENSMUSG00000112121	49	178	1.851	3.6E-21	C230072F16Rik
ENSMUSG00000020190	12097	25285	1.063	3.9E-21	Mknk2
ENSMUSG00000038332	7889	17231	1.127	4.0E-21	Sesn1
ENSMUSG00000026473	25968	65352	1.331	4.7E-21	Glul
ENSMUSG00000002346	3436	1683	-1.029	4.8E-21	Slc25a42
ENSMUSG00000028307	603	2545	2.078	6.1E-21	Aldob
ENSMUSG00000040128	3130	8126	1.376	6.8E-21	Pnrc1
ENSMUSG00000025150	634	232	-1.451	7.5E-21	Cbr2
ENSMUSG00000073481	1112	1876	0.755	8.4E-21	02. Mrz
ENSMUSG00000018669	895	1574	0.813	1.1E-20	Cdk5rap3
ENSMUSG00000029581	800	246	-1.704	1.1E-20	Fscn1
ENSMUSG00000027435	2667	1301	-1.036	1.3E-20	Cd93
ENSMUSG00000026042	1286	631	-1.027	1.5E-20	Col5a2
ENSMUSG00000026308	1904	933	-1.030	1.7E-20	Klhl30
ENSMUSG00000096586	12	170	3.827	1.9E-20	Gm22918
ENSMUSG00000101904	25	238	3.240	1.9E-20	Gm29427
ENSMUSG00000093577	99	357	1.851	2.1E-20	Gm20632
ENSMUSG00000032523	3010	1901	-0.664	2.4E-20	Hhatl
ENSMUSG00000079227	182	9	-4.395	2.4E-20	Ccr5
ENSMUSG00000029131	2432	3989	0.714	2.7E-20	Dnajb6
ENSMUSG00000024440	557	113	-2.303	3.0E-20	Pcdh12
ENSMUSG00000038393	30662	14579	-1.073	3.2E-20	Txnip
ENSMUSG00000029718	1503	676	-1.152	4.4E-20	Pcolce
ENSMUSG00000034209	206	54	-1.954	4.6E-20	Rasl10a
ENSMUSG00000034135	3649	7647	1.067	5.1E-20	Sik3
ENSMUSG00000044948	651	1522	1.224	5.2E-20	Cfap43
ENSMUSG00000020572	9298	15693	0.755	6.1E-20	Nampt
ENSMUSG00000003948	613	219	-1.480	6.8E-20	Mmd
ENSMUSG00000022895	2789	1235	-1.176	8.6E-20	Ets2
ENSMUSG00000020919	2997	4945	0.722	1.0E-19	Stat5b
ENSMUSG00000049092	205	512	1.328	1.1E-19	Gpr137c
ENSMUSG00000049287	982	556	-0.822	1.1E-19	Iba57
ENSMUSG00000014599	1396	530	-1.395	1.2E-19	Csf1
ENSMUSG00000027999	1895	3536	0.900	1.5E-19	Pla2g12a
ENSMUSG00000019539	581	227	-1.351	2.3E-19	Rcn3
ENSMUSG00000034205	437	124	-1.818	2.3E-19	Loxl2
ENSMUSG00000026856	311	105	-1.563	2.8E-19	Dolpp1

ENSMUSG00000062077	5644	10544	0.901	3.4E-19	Trim54
ENSMUSG00000052713	410	175	-1.225	3.5E-19	Zfp608
ENSMUSG00000072612	17	113	2.772	4.8E-19	Gm10382
ENSMUSG00000028124	936	2336	1.320	5.0E-19	Gclm
ENSMUSG00000025006	16455	33103	1.008	5.2E-19	Sorbs1
ENSMUSG00000059408	139	28	-2.300	5.3E-19	Mrgprh
ENSMUSG00000031799	3122	1775	-0.815	7.6E-19	Tpm4
ENSMUSG00000021255	1275	2207	0.792	1.5E-18	Esrrb
ENSMUSG00000048371	1445	596	-1.279	1.8E-18	Pdp2
ENSMUSG00000112734	1166	2785	1.255	2.2E-18	Gm47644
ENSMUSG00000020530	2442	3771	0.627	2.2E-18	Ggnbp2
ENSMUSG00000109029	6	103	3.988	2.8E-18	Gm44717
ENSMUSG00000041143	437	798	0.872	2.8E-18	Tmco4
ENSMUSG00000087242	17	259	3.905	3.0E-18	C78197
ENSMUSG00000032802	686	2723	1.988	3.2E-18	Srxn1
ENSMUSG00000049940	1655	2699	0.706	3.4E-18	Pgrmc2
ENSMUSG00000040485	105	453	2.095	3.7E-18	Lrrc52
ENSMUSG00000101970	711	1349	0.924	3.8E-18	1810026B05Rik
ENSMUSG00000021690	2306	4879	1.081	5.6E-18	Jmy
ENSMUSG00000063445	119	372	1.637	5.6E-18	Nmral1
ENSMUSG00000034472	177	518	1.542	5.8E-18	Rasd2
ENSMUSG00000004730	359	118	-1.610	7.0E-18	Adgre1
ENSMUSG00000040435	890	2253	1.340	7.1E-18	Ppp1r15a
ENSMUSG00000038178	673	1658	1.300	7.4E-18	Slc43a2
ENSMUSG00000036446	1759	540	-1.702	8.0E-18	Lum
ENSMUSG00000024352	136	310	1.193	9.1E-18	Spata24
ENSMUSG00000022474	635	1150	0.856	9.6E-18	Pmm1
ENSMUSG00000024978	6244	2905	-1.104	1.4E-17	Gpam
ENSMUSG00000043831	692	378	-0.870	1.5E-17	Lysmd4
ENSMUSG00000035764	639	366	-0.804	1.7E-17	Fbxo45
ENSMUSG00000024914	5694	10793	0.923	1.8E-17	Drap1
ENSMUSG00000030306	6384	13338	1.063	2.3E-17	Tmtc1
ENSMUSG00000034842	11345	19897	0.810	2.6E-17	Art3
ENSMUSG00000027488	3187	6672	1.066	2.7E-17	Snta1
ENSMUSG00000023885	1114	2058	0.883	2.7E-17	Thbs2
ENSMUSG00000064343	243	71	-1.792	3.2E-17	mt-Tq
ENSMUSG00000020130	1230	2354	0.937	4.0E-17	Tbc1d15
ENSMUSG00000028369	532	222	-1.256	4.3E-17	Svep1
ENSMUSG00000048486	8097	5141	-0.655	4.6E-17	Fitm2
ENSMUSG00000035121	89	262	1.553	4.8E-17	Neil2
ENSMUSG00000005534	6787	13742	1.018	4.9E-17	Insr
ENSMUSG00000035606	465	137	-1.765	5.0E-17	Ky
ENSMUSG00000023277	7150	14434	1.013	6.7E-17	Twf2
ENSMUSG00000006301	2401	4971	1.049	7.0E-17	Tmbim1
ENSMUSG00000003382	1166	1813	0.637	7.0E-17	Etv3
ENSMUSG00000033446	303	129	-1.229	7.6E-17	Lpar6
ENSMUSG00000074582	2782	4624	0.733	8.6E-17	Arfgef2
ENSMUSG00000022897	1991	3279	0.719	9.4E-17	Dyrk1a
ENSMUSG00000032035	2107	857	-1.298	1.0E-16	Ets1
ENSMUSG00000033308	391	753	0.947	1.0E-16	Dpyd
ENSMUSG00000032812	1193	717	-0.733	1.1E-16	Arap1
ENSMUSG00000115125	89	435	2.294	1.1E-16	Gm6852

ENSMUSG00000032245	727	1168	0.682	1.3E-16	Cln6
ENSMUSG00000018143	1743	3830	1.135	1.4E-16	Mafk
ENSMUSG00000024423	755	2558	1.759	1.6E-16	Impact
ENSMUSG00000038028	2389	4224	0.823	2.2E-16	Tigar
ENSMUSG00000027408	202	62	-1.718	2.2E-16	Cpxm1
ENSMUSG00000055541	90	14	-2.706	2.4E-16	Lair1
ENSMUSG00000028954	1271	2071	0.702	2.6E-16	Nub1
ENSMUSG00000021456	854	332	-1.364	2.7E-16	Fbp2
ENSMUSG00000041633	438	170	-1.370	2.9E-16	Kctd12b
ENSMUSG00000018932	2190	3512	0.681	2.9E-16	Map2k3
ENSMUSG00000040213	976	1602	0.716	3.1E-16	Kyat3
ENSMUSG00000062270	5302	8323	0.650	3.3E-16	Morf4l1
ENSMUSG00000015365	2054	3656	0.833	4.0E-16	Mov10l1
ENSMUSG00000015340	307	121	-1.346	4.3E-16	Cybb
ENSMUSG00000058297	878	2208	1.330	4.3E-16	Spock2
ENSMUSG00000056708	886	2535	1.516	4.4E-16	Ier5
ENSMUSG00000021273	1507	2645	0.812	4.6E-16	Fdft1
ENSMUSG00000014195	2992	4741	0.663	4.7E-16	Dnajc7
ENSMUSG00000039891	28227	42733	0.598	5.5E-16	Txlnb
ENSMUSG00000045817	1316	777	-0.762	7.1E-16	Zfp36l2
ENSMUSG00000022340	261	82	-1.671	8.2E-16	Sybu
ENSMUSG00000040451	2235	4642	1.054	9.4E-16	Sgms1
ENSMUSG00000075033	41	135	1.693	1.0E-15	Nxpe3
ENSMUSG00000078566	5897	14504	1.298	1.0E-15	Bnip3
ENSMUSG00000029804	1750	2952	0.754	1.2E-15	Herc3
ENSMUSG00000025190	31243	58065	0.894	1.7E-15	Got1
ENSMUSG00000028986	1577	3304	1.068	1.8E-15	Klhl7
ENSMUSG00000029657	1620	768	-1.078	1.9E-15	Hsph1
ENSMUSG00000069516	5355	2463	-1.120	1.9E-15	Lyz2
ENSMUSG00000028479	610	1193	0.966	2.0E-15	Gne
ENSMUSG00000015312	462	1143	1.303	2.1E-15	Gadd45b
ENSMUSG00000054435	674	331	-1.029	2.1E-15	Gimap4
ENSMUSG00000023886	2268	1112	-1.029	2.1E-15	Smoc2
ENSMUSG00000040414	891	1557	0.804	2.5E-15	Slc25a28
ENSMUSG00000022665	3063	1218	-1.330	2.5E-15	Ccdc80
ENSMUSG00000019558	9219	13906	0.593	2.7E-15	Slc6a8
ENSMUSG00000040666	5881	2828	-1.056	2.7E-15	Sh3bgr
ENSMUSG00000025193	492	822	0.743	2.9E-15	Cutc
ENSMUSG00000066357	562	302	-0.892	2.9E-15	Wdr6
ENSMUSG00000097357	265	63	-2.070	3.4E-15	Gm16793
ENSMUSG00000001785	672	1062	0.660	3.6E-15	Pwp1
ENSMUSG00000047714	3683	5241	0.509	3.7E-15	Ppp1r2
ENSMUSG00000104263	163	31	-2.376	3.7E-15	9430062P05Rik
ENSMUSG00000027544	665	237	-1.488	3.9E-15	Nfatc2
ENSMUSG00000100876	33	129	1.975	4.1E-15	2810454H06Rik
ENSMUSG00000030513	2388	971	-1.299	4.3E-15	Pcsk6
ENSMUSG00000015342	407	165	-1.314	4.3E-15	Xk
ENSMUSG00000038132	8859	13992	0.659	4.5E-15	Rbm24
ENSMUSG00000045664	386	153	-1.338	4.8E-15	Cdc42ep2
ENSMUSG00000033863	5981	8936	0.579	4.9E-15	Klf9
ENSMUSG00000022863	186	450	1.280	5.0E-15	Btg3
ENSMUSG00000015568	221930	382831	0.787	5.0E-15	Lpl

ENSMUSG00000034640	528	900	0.766	5.1E-15	Tiparp
ENSMUSG00000046329	1646	1039	-0.664	5.8E-15	Slc25a23
ENSMUSG00000045636	6789	4109	-0.725	5.8E-15	Mtus1
ENSMUSG00000097451	314	142	-1.146	5.9E-15	Rian
ENSMUSG00000050592	549	196	-1.483	6.0E-15	Fam78a
ENSMUSG00000022041	62	5	-3.506	6.2E-15	Chrna2
ENSMUSG00000031382	5264	10491	0.995	6.3E-15	Asb11
ENSMUSG00000052316	902	255	-1.826	6.9E-15	Lrrc15
ENSMUSG00000031965	3314	6422	0.955	7.3E-15	Tbx20
ENSMUSG00000100257	7	94	3.867	7.6E-15	C4bp-ps1
ENSMUSG00000022129	188	560	1.569	8.7E-15	Dct
ENSMUSG00000042770	268	550	1.036	8.7E-15	Hebp1
ENSMUSG00000053483	646	377	-0.778	9.3E-15	Usp21
ENSMUSG00000108354	7	76	3.360	9.5E-15	4931431B13Rik
ENSMUSG00000030847	6678	14428	1.111	9.6E-15	Bag3
ENSMUSG00000099696	19	154	3.024	1.0E-14	2900052N01Rik
ENSMUSG00000025337	1509	2246	0.575	1.0E-14	Sbds
ENSMUSG00000006784	22	134	2.631	1.1E-14	Ttc25
ENSMUSG00000022139	6715	10829	0.689	1.2E-14	Mbnl2
ENSMUSG00000039450	292	591	1.022	1.2E-14	Dcxr
ENSMUSG00000074794	1766	4603	1.383	1.3E-14	Arrdc3
ENSMUSG00000035629	1841	1065	-0.791	1.4E-14	Rubcn
ENSMUSG00000070476	508	220	-1.204	1.4E-14	Fam217b
ENSMUSG00000004939	440	1605	1.866	1.4E-14	Nmrk2
ENSMUSG00000087497	185	389	1.076	1.5E-14	2810001G20Rik
ENSMUSG00000026921	3054	1779	-0.780	1.6E-14	Egfl7
ENSMUSG00000037966	1705	3280	0.943	1.7E-14	Ninj1
ENSMUSG00000036890	383	675	0.823	1.7E-14	Gtdc1
ENSMUSG00000026812	1942	3117	0.683	1.9E-14	Tsc1
ENSMUSG00000035530	16893	26567	0.653	1.9E-14	Eif1
ENSMUSG00000031304	138	36	-1.930	1.9E-14	Il2rg
ENSMUSG00000036334	107	30	-1.850	2.0E-14	Igsf10
ENSMUSG00000106734	258	114	-1.186	2.0E-14	Gm20559
ENSMUSG00000037685	1748	3014	0.786	2.0E-14	Atp8a1
ENSMUSG00000050017	1228	1826	0.572	2.0E-14	Pitpnb
ENSMUSG00000037214	241	105	-1.202	2.1E-14	Thap1
ENSMUSG00000044442	918	1404	0.612	2.1E-14	N6amt1
ENSMUSG00000001518	484	783	0.693	2.2E-14	Itfg2
ENSMUSG00000078532	245	89	-1.481	2.3E-14	Nkain1
ENSMUSG00000019851	4908	7065	0.526	2.5E-14	Perp
ENSMUSG00000020435	1140	2106	0.884	2.8E-14	Osbp2
ENSMUSG00000022150	1184	506	-1.227	2.8E-14	Dab2
ENSMUSG00000004937	2896	4132	0.513	2.8E-14	Sgta
ENSMUSG00000024095	1065	1927	0.855	2.8E-14	Hnrnpll
ENSMUSG00000053460	746	355	-1.071	2.8E-14	Ggcx
ENSMUSG00000020990	410	901	1.139	2.9E-14	Cdkl1
ENSMUSG00000038074	422	227	-0.890	2.9E-14	Fkbp14
ENSMUSG00000068874	4010	6000	0.582	3.3E-14	Selenbp1
ENSMUSG00000043719	338	114	-1.573	3.3E-14	Col6a6
ENSMUSG00000070369	16	104	2.723	3.7E-14	Itgad
ENSMUSG00000055912	1004	469	-1.100	3.9E-14	Tmem150a
ENSMUSG00000030087	3944	8611	1.126	4.0E-14	Klf15

ENSMUSG00000032334	1176	583	-1.012	4.0E-14	Loxl1
ENSMUSG00000023074	1362	2495	0.874	4.0E-14	Mospd1
ENSMUSG00000042148	2470	3964	0.682	4.1E-14	Cox10
ENSMUSG00000109696	64	173	1.425	4.2E-14	Gm36670
ENSMUSG00000023959	12215	23401	0.938	4.2E-14	Clic5
ENSMUSG00000020914	68	4	-3.864	4.8E-14	Top2a
ENSMUSG00000033228	2136	3109	0.542	5.2E-14	Scaf11
ENSMUSG00000023259	436	190	-1.196	5.2E-14	Slc26a6
ENSMUSG00000020250	2098	3868	0.883	5.6E-14	Txnrd1
ENSMUSG00000032263	1803	2913	0.692	6.3E-14	Bckdhb
ENSMUSG00000026548	97	24	-2.021	6.3E-14	Slamf9
ENSMUSG00000026837	1714	784	-1.128	6.6E-14	Col5a1
ENSMUSG00000010095	1237	2925	1.241	6.9E-14	Slc3a2
ENSMUSG00000047022	100	229	1.197	7.4E-14	Mipol1
ENSMUSG00000047242	389	632	0.701	7.4E-14	Taf9b
ENSMUSG00000020088	7073	10924	0.627	8.1E-14	Sar1a
ENSMUSG00000031880	4286	1453	-1.561	8.2E-14	Rrad
ENSMUSG00000002769	193	730	1.921	8.2E-14	Gnmt
ENSMUSG00000020034	6222	10673	0.779	8.5E-14	Tcp11l2
ENSMUSG00000058135	6248	10790	0.788	9.0E-14	Gstm1
ENSMUSG00000108626	53	203	1.943	9.1E-14	Gm40457
ENSMUSG00000054717	506	1006	0.994	9.1E-14	Hmgb2
ENSMUSG00000031700	1557	831	-0.906	9.5E-14	Gpt2
ENSMUSG00000043639	9385	15427	0.717	1.0E-13	Rbm20
ENSMUSG00000022817	4283	6628	0.629	1.0E-13	Itgb5
ENSMUSG00000028869	1230	1853	0.589	1.0E-13	Gnl2
ENSMUSG00000038451	89	233	1.396	1.1E-13	Spsb2
ENSMUSG00000069844	805	475	-0.759	1.1E-13	Sco1
ENSMUSG00000031875	506	257	-0.980	1.2E-13	Cmtm3
ENSMUSG00000029084	487	238	-1.037	1.2E-13	Cd38
ENSMUSG00000002981	3524	5139	0.544	1.2E-13	Clptm1
ENSMUSG00000105442	1	91	5.977	1.2E-13	Gm42614
ENSMUSG00000031698	25921	42639	0.718	1.2E-13	Mylk3
ENSMUSG00000086881	56	178	1.669	1.2E-13	Gm13594
ENSMUSG00000019996	869	1538	0.823	1.3E-13	Map7
ENSMUSG00000020780	2548	3924	0.622	1.3E-13	Srp68
ENSMUSG00000025980	13892	8428	-0.721	1.3E-13	Hspd1
ENSMUSG00000061751	778	1436	0.882	1.4E-13	Kalrn
ENSMUSG00000030110	183	62	-1.566	1.4E-13	Ret
ENSMUSG00000086451	161	364	1.177	1.4E-13	4933431K23Rik
ENSMUSG00000027204	2162	927	-1.222	1.4E-13	Fbn1
ENSMUSG00000074063	144	340	1.232	1.4E-13	Osgin1
ENSMUSG00000000317	551	255	-1.110	1.5E-13	Bcl6b
ENSMUSG00000042870	1854	3296	0.829	1.6E-13	Tom1
ENSMUSG00000026932	1225	718	-0.773	1.6E-13	Nacc2
ENSMUSG00000030116	880	469	-0.909	1.6E-13	Mfap5
ENSMUSG00000027533	1191	669	-0.831	1.7E-13	Fabp5
ENSMUSG00000038990	665	285	-1.227	1.7E-13	Cables2
ENSMUSG00000038612	8280	13681	0.724	1.8E-13	Mcl1
ENSMUSG00000036904	281	126	-1.161	1.9E-13	Fzd8
ENSMUSG00000050708	3942	9885	1.326	1.9E-13	Ftl1
ENSMUSG00000040350	1019	303	-1.751	1.9E-13	Trim7

ENSMUSG00000032816	734	356	-1.048	2.1E-13	Igdcc4
ENSMUSG00000071984	517	136	-1.934	2.2E-13	Fndc1
ENSMUSG00000016194	468	1063	1.183	2.2E-13	Hsd11b1
ENSMUSG00000034295	12474	17365	0.477	2.2E-13	Fhod3
ENSMUSG00000007655	7081	3648	-0.957	2.5E-13	Cav1
ENSMUSG00000021256	397	188	-1.079	2.7E-13	Vash1
ENSMUSG00000058351	502	1107	1.145	2.7E-13	Smim4
ENSMUSG00000045896	1955	3150	0.689	2.8E-13	Paip2b
ENSMUSG00000021686	1362	2107	0.628	2.9E-13	Ap3b1
ENSMUSG00000055296	4517	9499	1.072	2.9E-13	Tmem245
ENSMUSG00000059552	519	237	-1.131	2.9E-13	Trp53
ENSMUSG00000099342	5	47	3.184	2.9E-13	Gm18180
ENSMUSG00000091049	6	47	3.042	3.1E-13	Gm1979
ENSMUSG00000023861	5208	8036	0.626	3.4E-13	Mpc1
ENSMUSG00000103983	109	24	-2.174	3.4E-13	Gm20045
ENSMUSG00000097242	116	346	1.569	3.5E-13	Gm16907
ENSMUSG00000045838	1581	869	-0.864	3.7E-13	Ccdc9b
ENSMUSG00000031586	4799	7377	0.620	3.8E-13	Rbpms
ENSMUSG00000031389	90	24	-1.951	3.8E-13	Arhgap4
ENSMUSG00000053470	1137	1838	0.692	3.9E-13	Kdm3a
ENSMUSG00000064202	409	691	0.758	4.0E-13	4430402I18Rik
ENSMUSG00000027255	1388	2042	0.556	4.3E-13	Arfgap2
ENSMUSG00000018566	14075	23716	0.753	4.6E-13	Slc2a4
ENSMUSG00000027381	704	1337	0.922	4.6E-13	Bcl2l11
ENSMUSG00000039361	6048	12065	0.996	4.6E-13	Picalm
ENSMUSG00000055322	53232	105058	0.981	4.9E-13	Tns1
ENSMUSG00000017817	19579	30051	0.618	5.0E-13	Jph2
ENSMUSG00000038816	1173	3533	1.592	5.0E-13	Ctnnal1
ENSMUSG00000003809	2690	3836	0.512	5.0E-13	Gcdh
ENSMUSG00000035021	321	772	1.265	5.0E-13	Baz1a
ENSMUSG00000036144	373	186	-0.997	5.2E-13	Meox2
ENSMUSG00000099137	86	15	-2.545	5.3E-13	Gm10603
ENSMUSG00000108414	238	441	0.884	5.4E-13	Snhg1
ENSMUSG00000074622	728	307	-1.248	5.7E-13	Mafb
ENSMUSG00000040705	75	10	-2.865	5.8E-13	A930016O22Rik
ENSMUSG00000024853	4979	7466	0.584	5.9E-13	Sf3b2
ENSMUSG00000028427	1402	503	-1.480	6.0E-13	Aqp7
ENSMUSG00000035342	3129	5315	0.764	6.1E-13	Lzts2
ENSMUSG00000036545	952	519	-0.874	6.2E-13	Adamts2
ENSMUSG00000041313	2194	4179	0.930	6.3E-13	Slc7a1
ENSMUSG00000027293	15403	23217	0.592	6.4E-13	Ehd4
ENSMUSG00000022965	5040	2446	-1.044	6.5E-13	Ifngr2
ENSMUSG00000022358	8645	17828	1.044	6.6E-13	Fbxo32
ENSMUSG00000046675	240	452	0.913	6.9E-13	Tmem251
ENSMUSG00000039031	529	233	-1.178	6.9E-13	Arhgap18
ENSMUSG00000020532	688	1523	1.150	7.0E-13	Acaca
ENSMUSG00000020101	1023	570	-0.846	7.3E-13	Vsir
ENSMUSG00000052085	523	222	-1.240	7.6E-13	Dock8
ENSMUSG00000036104	2160	2958	0.453	8.1E-13	Rab3gap1
ENSMUSG00000068196	771	421	-0.874	8.2E-13	Col8a1
ENSMUSG00000086784	1880	2725	0.536	8.4E-13	Isoc2a
ENSMUSG00000033361	499	218	-1.203	8.7E-13	Prrg3

ENSMUSG00000037573	1713	2890	0.754	9.7E-13	Tob1
ENSMUSG00000062991	20	127	2.681	9.8E-13	Nrg1
ENSMUSG00000029833	1136	1640	0.529	9.9E-13	Trim24
ENSMUSG00000082676	7	59	2.974	1.0E-12	Gm11843
ENSMUSG00000024654	2169	3303	0.607	1.0E-12	Asrgl1
ENSMUSG00000027602	13738	20460	0.575	1.0E-12	Map1lc3a
ENSMUSG00000072568	249	90	-1.472	1.1E-12	Fam84b
ENSMUSG00000046792	924	1364	0.560	1.2E-12	Zfp787
ENSMUSG00000020520	524	285	-0.884	1.2E-12	Galnt10
ENSMUSG00000052336	82	7	-3.438	1.3E-12	Cx3cr1
ENSMUSG00000031617	514	817	0.667	1.3E-12	Tmem184c
ENSMUSG00000068290	2518	3889	0.627	1.3E-12	Ddrgk1
ENSMUSG00000030711	1811	3699	1.029	1.3E-12	Sult1a1
ENSMUSG00000038457	161	52	-1.621	1.3E-12	Tmem255b
ENSMUSG00000019082	2022	1129	-0.841	1.5E-12	Slc25a22
ENSMUSG00000031701	8241	13030	0.661	1.5E-12	Dnaja2
ENSMUSG00000079523	1001	437	-1.196	1.5E-12	Tmsb10
ENSMUSG00000047419	23939	33847	0.500	1.5E-12	Cmya5
ENSMUSG00000021892	2131	3518	0.723	1.5E-12	Sh3bp5
ENSMUSG00000105622	5	224	5.626	1.7E-12	Gm42615
ENSMUSG00000033715	284	109	-1.387	1.7E-12	Akr1c14
ENSMUSG00000032011	229	95	-1.273	1.7E-12	Thy1
ENSMUSG00000026712	1222	565	-1.114	1.7E-12	Mrc1
ENSMUSG00000033610	2736	4608	0.752	1.8E-12	Pank1
ENSMUSG00000029521	140	50	-1.497	1.8E-12	Chek2
ENSMUSG00000035944	1064	1795	0.754	1.9E-12	Ttc38
ENSMUSG00000029723	4492	2841	-0.661	1.9E-12	Tsc22d4
ENSMUSG00000038695	2049	1324	-0.631	2.0E-12	Josd2
ENSMUSG00000039886	497	891	0.843	2.0E-12	Tmem120a
ENSMUSG00000029163	939	477	-0.979	2.0E-12	Emilin1
ENSMUSG00000060639	74	351	2.249	2.2E-12	Hist1h4i
ENSMUSG00000022816	3262	1661	-0.974	2.2E-12	Fstl1
ENSMUSG00000035697	424	136	-1.641	2.3E-12	Arhgap45
