# Supplementary material for: The conjugation-resistant bile acid norUDCA cures liver fibrosis but impairs systemic energy metabolism
Source: Mol Metab. 2026 Apr 2;107:102363. doi: 10.1016/j.molmet.2026.102363 (PMC13096911; doi:10.1016/j.molmet.2026.102363)
Supplement: Multimedia component 3 [file mmc3.pdf]

gene_id	Control	norUDCA	log2FoldChange	padj	gene_name
ENSMUSG00000040016	1271	122	-3.384	2.53E-86	Ptger3
ENSMUSG00000031489	6355	376	-4.080	2.60E-81	Adrb3
ENSMUSG00000028339	1654	125	-3.723	3.21E-67	Col15a1
ENSMUSG00000025533	373	3705	3.313	3.73E-65	Asl
ENSMUSG00000020427	500	3430	2.779	9.15E-63	Igfbp3
ENSMUSG00000061740	2244	7423	1.726	2.93E-61	Cyp2d22
ENSMUSG00000049971	106	788	2.885	4.41E-57	Glt1d1
ENSMUSG00000068220	11583	3507	-1.724	1.02E-56	Lgals1
ENSMUSG00000048756	451	1518	1.751	3.10E-56	Foxo3
ENSMUSG00000112129	461	2627	2.510	3.41E-52	Pbld1
ENSMUSG00000040618	736	2932	1.994	1.58E-41	Pck2
ENSMUSG00000020680	1489	2967	0.995	1.98E-41	Taf15
ENSMUSG00000046794	7339	1079	-2.766	1.98E-41	Ppp1r3b
ENSMUSG00000045193	808	2439	1.593	9.83E-41	Cirbp
ENSMUSG00000026489	28347	68996	1.283	5.70E-40	Coq8a
ENSMUSG00000025355	977	333	-1.552	5.70E-40	Mmp19
ENSMUSG00000027597	586	2353	2.004	3.60E-38	Ahcy
ENSMUSG00000029675	889	2014	1.179	1.16E-36	Eln
ENSMUSG00000046275	5266	817	-2.688	1.25E-36	Tusc5
ENSMUSG00000073700	803	2520	1.651	1.61E-36	Klhl21
ENSMUSG00000024990	3048	494	-2.625	1.74E-36	Rbp4
ENSMUSG00000021024	3986	9148	1.199	8.05E-36	Psma6
ENSMUSG00000031770	2106	11880	2.496	2.20E-35	Herpud1
ENSMUSG00000026043	8439	769	-3.456	2.79E-35	Col3a1
ENSMUSG00000073758	186	692	1.896	3.40E-35	Sh3d21
ENSMUSG00000025340	708	2109	1.576	5.37E-35	Rabgef1
ENSMUSG00000061742	14	295	4.372	1.36E-34	Slc22a12
ENSMUSG00000045917	707	2629	1.894	2.73E-34	Tmem268
ENSMUSG00000064215	28796	17082	-0.753	9.42E-34	Ifi27
ENSMUSG00000036528	244	1143	2.230	3.51E-33	Ppfibp2
ENSMUSG00000020472	299	747	1.324	5.01E-33	Zkscan17
ENSMUSG00000018593	19174	6133	-1.644	8.20E-33	Sparc
ENSMUSG00000029370	263	921	1.811	1.15E-31	Rassf6
ENSMUSG00000020432	2843	6811	1.261	1.74E-31	Tcn2
ENSMUSG00000020034	659	2180	1.725	2.78E-31	Tcp11l2
ENSMUSG00000031026	43	348	3.042	4.61E-31	Trim66
ENSMUSG00000026626	26261	10943	-1.263	5.79E-31	Ppp2r5a
ENSMUSG00000078429	2845	5280	0.892	1.49E-30	Ctdsp2
ENSMUSG00000030161	5045	12788	1.342	3.38E-30	Gabarapl1
ENSMUSG00000025202	17	165	3.287	8.12E-30	Scd3
ENSMUSG00000028701	1019	259	-1.978	1.04E-29	Lurap1
ENSMUSG00000022548	145	941	2.690	1.44E-29	Apod
ENSMUSG00000022947	91	603	2.732	2.26E-29	Cbr3
ENSMUSG00000039530	286	1154	2.013	5.16E-29	Tusc3
ENSMUSG00000025792	1106	329	-1.748	5.16E-29	Slc25a10
ENSMUSG00000031980	73	1051	3.837	5.24E-29	Agt
ENSMUSG00000079017	4560	613	-2.896	5.80E-29	Ifi27l2a
ENSMUSG00000040713	1810	3478	0.942	8.85E-29	Creg1
ENSMUSG00000020534	819	2018	1.302	1.37E-28	Shmt1
ENSMUSG00000020836	597	1938	1.701	2.65E-28	Coro6
ENSMUSG00000020547	613	2085	1.765	5.26E-28	Bzw2

ENSMUSG00000022665	1847	430	-2.102	5.26E-28	Ccdc80
ENSMUSG00000049241	723	101	-2.839	6.05E-28	Hcar1
ENSMUSG00000048489	1297	13323	3.361	1.04E-27	Depp1
ENSMUSG00000046027	766	1950	1.347	1.04E-27	Stard5
ENSMUSG00000019853	176	26	-2.786	3.12E-27	Hebp2
ENSMUSG00000033416	3255	8374	1.363	3.94E-27	Gucd1
ENSMUSG00000003948	6815	1991	-1.775	5.72E-27	Mmd
ENSMUSG00000022299	1468	5730	1.964	1.09E-26	Slc25a32
ENSMUSG00000039395	112	511	2.185	1.37E-26	Mreg
ENSMUSG00000029669	4038	1987	-1.023	1.89E-26	Tspan12
ENSMUSG00000053411	623	1676	1.427	1.67E-25	Cbx7
ENSMUSG00000002058	380	113	-1.752	5.48E-25	Unc119
ENSMUSG00000033444	1036	1994	0.945	8.53E-25	Specc1l
ENSMUSG00000027533	2650	648	-2.032	9.69E-25	Fabp5
ENSMUSG00000055254	4844	2678	-0.855	1.45E-24	Ntrk2
ENSMUSG00000022853	7962	50617	2.668	1.69E-24	Ehhadh
ENSMUSG00000019326	5977	2339	-1.354	2.52E-24	Aoc3
ENSMUSG00000036206	250	1339	2.420	2.85E-24	Sh3bp4
ENSMUSG00000067279	2342	348	-2.750	3.04E-24	Ppp1r3c
ENSMUSG00000058297	114	370	1.699	3.27E-24	Spock2
ENSMUSG00000067786	556	42	-3.734	4.31E-24	Nnat
ENSMUSG00000048787	1754	624	-1.492	5.02E-24	Dcun1d3
ENSMUSG00000020848	14	132	3.233	9.20E-24	Doc2b
ENSMUSG00000029135	461	3050	2.727	1.08E-23	Fosl2
ENSMUSG00000073460	386	89	-2.118	1.09E-23	Pnldc1
ENSMUSG00000051314	163	21	-2.989	1.14E-23	Ffar2
ENSMUSG00000023074	815	1535	0.913	4.09E-23	Mospd1
ENSMUSG00000000628	11650	3475	-1.745	4.92E-23	Hk2
ENSMUSG00000027712	8539	4271	-1.000	5.60E-23	Anxa5
ENSMUSG00000070476	533	198	-1.428	6.21E-23	Fam217b
ENSMUSG00000047181	176	597	1.766	1.08E-22	Samd14
ENSMUSG00000006800	1523	718	-1.084	1.11E-22	Sulf2
ENSMUSG00000038546	1666	5237	1.652	1.27E-22	Ranbp9
ENSMUSG00000042569	2711	1564	-0.793	1.63E-22	Dhrs7b
ENSMUSG00000051483	2003	5723	1.515	2.15E-22	Cbr1
ENSMUSG00000050373	1197	3758	1.650	2.55E-22	Snx21
ENSMUSG00000024646	4055	8699	1.101	4.35E-22	Cyb5a
ENSMUSG00000031812	5665	12144	1.100	5.79E-22	Map1lc3b
ENSMUSG00000042622	230	1190	2.373	6.17E-22	Maff
ENSMUSG00000036478	2371	6385	1.429	7.09E-22	Btg1
ENSMUSG00000034088	7525	13302	0.822	7.09E-22	Hdlbp
ENSMUSG00000061397	282	91	-1.626	1.52E-21	Krt79
ENSMUSG00000036833	708	2490	1.813	1.93E-21	Pnpla7
ENSMUSG00000032754	709	1979	1.480	2.00E-21	Slc8b1
ENSMUSG00000001506	2401	371	-2.695	2.94E-21	Col1a1
ENSMUSG00000061758	1367	761	-0.845	3.47E-21	Akr1b10
ENSMUSG00000032501	2396	426	-2.492	4.00E-21	Trib1
ENSMUSG00000003849	35	162	2.192	5.63E-21	Nqo1
ENSMUSG00000016194	6090	21507	1.820	5.63E-21	Hsd11b1
ENSMUSG00000054836	131	393	1.579	6.65E-21	Elp6
ENSMUSG00000041696	2025	432	-2.229	6.82E-21	Rasl12
ENSMUSG00000001138	463	885	0.933	9.90E-21	Cnnm3

ENSMUSG00000012187	178	40	-2.150	1.23E-20	Mogat1
ENSMUSG00000059201	139	1	-6.551	1.64E-20	Lep
ENSMUSG00000031710	115380	40801	-1.500	1.81E-20	Ucp1
ENSMUSG00000060181	312	724	1.211	2.04E-20	Slc35e3
ENSMUSG00000035283	708	70	-3.329	2.11E-20	Adrb1
ENSMUSG00000020774	1545	831	-0.895	5.49E-20	Aspa
ENSMUSG00000019139	551	1438	1.385	7.29E-20	Isyna1
ENSMUSG00000049999	785	167	-2.229	7.97E-20	Ppp1r3d
ENSMUSG00000040136	17	97	2.489	1.23E-19	Abcc8
ENSMUSG00000032401	1784	323	-2.464	1.34E-19	Lctl
ENSMUSG00000038393	25899	81459	1.653	1.44E-19	Txnip
ENSMUSG00000038332	462	1987	2.104	1.58E-19	Sesn1
ENSMUSG00000027313	70	324	2.207	1.88E-19	Chac1
ENSMUSG00000018846	4518	1656	-1.448	3.62E-19	Pank3
ENSMUSG00000036687	16	111	2.842	4.92E-19	Tmem184a
ENSMUSG00000001627	1142	3545	1.634	5.31E-19	Ifrd1
ENSMUSG00000070002	682	1504	1.141	5.31E-19	Ell
ENSMUSG00000044122	878	486	-0.852	7.74E-19	Proca1
ENSMUSG00000001270	714	2654	1.894	1.02E-18	Ckb
ENSMUSG00000020782	143	361	1.335	1.11E-18	Llgl2
ENSMUSG00000063410	730	1271	0.799	1.67E-18	Stk24
ENSMUSG00000019883	2026	771	-1.393	2.03E-18	Echdc1
ENSMUSG00000019577	58243	122046	1.067	2.04E-18	Pdk4
ENSMUSG00000045103	204	557	1.445	2.09E-18	Dmd
ENSMUSG00000044252	1796	3649	1.022	2.77E-18	Osbpl1a
ENSMUSG00000015852	86	15	-2.563	3.03E-18	Fcrls
ENSMUSG00000024030	308	1353	2.137	3.13E-18	Abcg1
ENSMUSG00000024011	320	49	-2.708	3.57E-18	Pi16
ENSMUSG00000044337	2239	400	-2.486	6.85E-18	Ackr3
ENSMUSG00000025515	82	405	2.304	7.61E-18	Muc2
ENSMUSG00000032531	1434	646	-1.151	9.59E-18	Amotl2
ENSMUSG00000098557	539	1492	1.469	9.79E-18	Kctd12
ENSMUSG00000020264	16590	8937	-0.892	9.97E-18	Slc36a2
ENSMUSG00000057137	465	1345	1.530	1.11E-17	Tmem140
ENSMUSG00000038807	200	755	1.917	1.28E-17	Rap1gap2
ENSMUSG00000021903	981	2645	1.430	1.59E-17	Galnt15
ENSMUSG00000025337	1297	2189	0.755	1.79E-17	Sbds
ENSMUSG00000019978	2812	1295	-1.118	3.43E-17	Epb41l2
ENSMUSG00000084128	40	237	2.578	3.68E-17	Esrp2
ENSMUSG00000020125	13	82	2.609	5.04E-17	Elane
ENSMUSG00000020241	2186	594	-1.880	6.22E-17	Col6a2
ENSMUSG00000025532	439	1262	1.524	6.98E-17	Crcp
ENSMUSG00000042726	1507	4087	1.439	7.38E-17	Trafd1
ENSMUSG00000059327	203	494	1.277	7.38E-17	Eda
ENSMUSG00000034659	2290	5196	1.182	7.42E-17	Tmem109
ENSMUSG00000038400	3358	1011	-1.731	8.40E-17	Pmepa1
ENSMUSG00000029096	1285	601	-1.095	9.47E-17	Htra3
ENSMUSG00000039518	204	50	-2.041	1.08E-16	Cdsn
ENSMUSG00000032290	620	1343	1.116	1.10E-16	Ptpn9
ENSMUSG00000025170	190	570	1.586	1.16E-16	Rab40b
ENSMUSG00000068874	1145	3048	1.412	1.48E-16	Selenbp1
ENSMUSG00000021559	580	1708	1.558	1.86E-16	Dapk1

ENSMUSG00000041769	1705	3250	0.931	2.29E-16	Ppp2r2d
ENSMUSG00000058258	474	127	-1.905	2.29E-16	Idi1
ENSMUSG00000033107	217	1031	2.246	2.33E-16	Rnf125
ENSMUSG00000025420	243	965	1.987	2.35E-16	Katnal2
ENSMUSG00000052373	97	395	2.026	2.36E-16	Mpp3
ENSMUSG00000059824	1452	258	-2.490	2.37E-16	Dbp
ENSMUSG00000022894	318	122	-1.379	2.60E-16	Adamts5
ENSMUSG00000025092	329	134	-1.302	3.66E-16	Hspa12a
ENSMUSG00000018217	1423	682	-1.061	5.24E-16	Pmp22
ENSMUSG00000025934	178	462	1.373	5.38E-16	Gsta3
ENSMUSG00000005846	905	1508	0.736	5.38E-16	Rsl1d1
ENSMUSG00000026483	1166	2530	1.117	8.55E-16	Fam129a
ENSMUSG00000030256	333	105	-1.661	8.82E-16	Bhlhe41
ENSMUSG00000051225	67	5	-3.753	8.92E-16	Fam83a
ENSMUSG00000070436	5254	1178	-2.157	1.05E-15	Serpinh1
ENSMUSG00000038456	133	385	1.535	1.23E-15	Dennd2a
ENSMUSG00000028967	629	2115	1.750	1.35E-15	Errfi1
ENSMUSG00000032724	187	449	1.261	1.89E-15	Abtb2
ENSMUSG00000019558	649	1500	1.208	1.89E-15	Slc6a8
ENSMUSG00000054263	1143	5858	2.357	2.25E-15	Lifr
ENSMUSG00000022863	280	696	1.314	2.53E-15	Btg3
ENSMUSG00000015980	290	93	-1.630	2.91E-15	Lrrc27
ENSMUSG00000001313	66	302	2.194	3.36E-15	Rnd2
ENSMUSG00000046447	215	527	1.293	3.79E-15	Camk2n1
ENSMUSG00000022024	1848	3226	0.804	4.14E-15	Sugt1
ENSMUSG00000037686	10554	29557	1.486	4.94E-15	Aspg
ENSMUSG00000064225	2734	990	-1.465	4.97E-15	Paqr9
ENSMUSG00000026837	956	287	-1.735	5.27E-15	Col5a1
ENSMUSG00000026773	24578	6324	-1.958	5.60E-15	Pfkfb3
ENSMUSG00000030849	232	696	1.580	5.75E-15	Fgfr2
ENSMUSG00000022999	489	1031	1.075	5.75E-15	Lmbr1l
ENSMUSG00000073481	2596	4299	0.728	5.75E-15	44257
ENSMUSG00000022330	29	138	2.227	6.49E-15	Osr2
ENSMUSG00000020430	1089	1726	0.665	7.03E-15	Pes1
ENSMUSG00000032018	1376	572	-1.265	7.89E-15	Sc5d
ENSMUSG00000027947	364	1407	1.948	8.67E-15	Il6ra
ENSMUSG00000039989	1390	269	-2.370	9.85E-15	Cbx4
ENSMUSG00000029752	1474	3075	1.060	1.11E-14	Asns
ENSMUSG00000090523	1430	637	-1.165	1.18E-14	Gypc
ENSMUSG00000043110	201	507	1.333	1.40E-14	Lrrn4
ENSMUSG00000029661	3726	867	-2.104	1.48E-14	Col1a2
ENSMUSG00000034957	22135	8862	-1.321	1.56E-14	Cebpa
ENSMUSG00000042678	13	85	2.657	1.67E-14	Myo15
ENSMUSG00000034903	727	1493	1.039	1.84E-14	Cobll1
ENSMUSG00000058135	2945	5178	0.815	1.99E-14	Gstm1
ENSMUSG00000055675	1238	520	-1.253	2.28E-14	Kbtbd11
ENSMUSG00000075701	847	1437	0.762	2.60E-14	Selenos
ENSMUSG00000021509	551	124	-2.144	2.98E-14	Slc25a48
ENSMUSG00000038803	1008	611	-0.723	3.01E-14	Ost4
ENSMUSG00000021025	1382	4122	1.576	3.09E-14	Nfkbia
ENSMUSG00000067235	5586	2560	-1.126	3.21E-14	H2-Q10
ENSMUSG00000033361	201	581	1.526	3.69E-14	Prrg3

ENSMUSG00000037788	262	512	0.964	3.97E-14	Vopp1
ENSMUSG00000004558	8794	16165	0.878	4.01E-14	Ndrg2
ENSMUSG00000018669	730	1307	0.840	4.24E-14	Cdk5rap3
ENSMUSG00000040414	815	1358	0.737	4.71E-14	Slc25a28
ENSMUSG00000027274	532	255	-1.059	5.09E-14	Mkks
ENSMUSG00000031298	126	460	1.864	6.27E-14	Adgrg2
ENSMUSG00000032078	471	804	0.770	6.82E-14	Zpr1
ENSMUSG00000090145	31	112	1.837	7.02E-14	Ugt1a6b
ENSMUSG00000020108	1623	17815	3.457	7.16E-14	Ddit4
ENSMUSG00000041734	254	590	1.217	7.66E-14	Kirrel
ENSMUSG00000027610	716	1561	1.123	7.78E-14	Gss
ENSMUSG00000020142	40	199	2.322	8.36E-14	Slc1a4
ENSMUSG00000027357	6970	11344	0.703	9.04E-14	Crls1
ENSMUSG00000066042	71	260	1.864	9.21E-14	Med18
ENSMUSG00000048126	2171	553	-1.974	1.02E-13	Col6a3
ENSMUSG00000049717	209	88	-1.243	1.07E-13	Lig4
ENSMUSG00000022747	702	1626	1.212	1.16E-13	St3gal6
ENSMUSG00000021360	266	911	1.775	1.52E-13	Gcnt2
ENSMUSG00000007659	1302	2515	0.949	1.62E-13	Bcl2l1
ENSMUSG00000023150	5975	23690	1.987	1.66E-13	Ivns1abp
ENSMUSG00000069456	4	79	4.197	1.83E-13	Rdh16
ENSMUSG00000021226	1780	5586	1.650	1.84E-13	Acot2
ENSMUSG00000053964	825	1765	1.098	1.85E-13	Lgals4
ENSMUSG00000022094	979	3153	1.687	1.85E-13	Slc39a14
ENSMUSG00000021242	11923	7284	-0.711	1.99E-13	Npc2
ENSMUSG00000005718	95	237	1.308	1.99E-13	Tfap4
ENSMUSG00000042628	690	1339	0.957	2.02E-13	Zfyve1
ENSMUSG00000052934	4148	18829	2.182	2.12E-13	Fbxo31
ENSMUSG00000045817	3932	1320	-1.575	2.32E-13	Zfp36l2
ENSMUSG00000039648	642	1083	0.755	2.34E-13	Kyat1
ENSMUSG00000037211	316	645	1.029	2.48E-13	Spry1
ENSMUSG00000038260	260	870	1.745	2.62E-13	Trpm4
ENSMUSG00000035172	1088	395	-1.461	2.78E-13	Plekhh3
ENSMUSG00000056413	320	89	-1.839	2.78E-13	Adap1
ENSMUSG00000089665	888	399	-1.156	2.82E-13	Fcor
ENSMUSG00000062373	2668	4345	0.704	3.53E-13	Tmem65
ENSMUSG00000034936	118	440	1.900	4.29E-13	Arl4d
ENSMUSG00000060981	420	130	-1.685	4.29E-13	Hist1h4h
ENSMUSG00000037606	503	1467	1.545	4.58E-13	Osbpl5
ENSMUSG00000079487	2062	3377	0.712	4.76E-13	Med12
ENSMUSG00000023176	850	333	-1.355	6.27E-13	Cpn2
ENSMUSG00000047875	165	412	1.320	6.63E-13	Gpr157
ENSMUSG00000069255	948	1665	0.813	6.65E-13	Dusp22
ENSMUSG00000027359	7816	15990	1.033	6.89E-13	Slc27a2
ENSMUSG00000025937	3089	1403	-1.138	7.07E-13	Lactb2
ENSMUSG00000028917	785	1652	1.075	7.12E-13	Plekhm2
ENSMUSG00000030934	1559	2875	0.883	7.83E-13	Oat
ENSMUSG00000038975	1594	2371	0.573	8.20E-13	Rabggtb
ENSMUSG00000031766	4	52	3.702	8.60E-13	Slc12a3
ENSMUSG00000002341	56	2	-5.025	9.02E-13	Ncan
ENSMUSG00000074918	900	438	-1.039	9.28E-13	Inafm2
ENSMUSG00000027875	148	693	2.229	1.00E-12	Hmgcs2

ENSMUSG00000074794	838	3090	1.882	1.01E-12	Arrdc3
ENSMUSG00000054499	618	1329	1.105	1.10E-12	Dedd2
ENSMUSG00000038422	284	88	-1.697	1.10E-12	Hdhd3
ENSMUSG00000025757	1301	465	-1.485	1.21E-12	Hspa4l
ENSMUSG00000006732	188	354	0.913	1.25E-12	Mettl1
ENSMUSG00000041577	5463	1799	-1.603	1.30E-12	Prelp
ENSMUSG00000089736	111	269	1.289	1.37E-12	Tgfbr3l
ENSMUSG00000039496	317	553	0.800	1.39E-12	Cdnf
ENSMUSG00000024440	292	67	-2.132	1.39E-12	Pcdh12
ENSMUSG00000028150	1148	3022	1.397	1.49E-12	Rorc
ENSMUSG00000028479	654	1162	0.830	1.69E-12	Gne
ENSMUSG00000021453	4203	13456	1.679	1.73E-12	Gadd45g
ENSMUSG00000002833	1009	1783	0.821	1.78E-12	Hdgfl2
ENSMUSG00000024972	3132	1161	-1.432	1.81E-12	Lgals12
ENSMUSG00000033768	54	203	1.912	1.82E-12	Nrxn2
ENSMUSG00000018474	2769	1104	-1.327	1.89E-12	Chd3
ENSMUSG00000083282	1414	2648	0.905	1.90E-12	Ctsf
ENSMUSG00000034614	215	1140	2.403	2.10E-12	Pik3ip1
ENSMUSG00000026576	337	746	1.148	2.10E-12	Atp1b1
ENSMUSG00000049303	429	142	-1.595	2.10E-12	Syt12
ENSMUSG00000016496	1876	738	-1.345	2.19E-12	Cd274
ENSMUSG00000055782	1595	566	-1.494	2.30E-12	Abcd2
ENSMUSG00000020023	1935	4153	1.101	2.69E-12	Tmcc3
ENSMUSG00000025068	2287	3761	0.718	2.79E-12	Gsto1
ENSMUSG00000072949	496	2463	2.312	3.06E-12	Acot1
ENSMUSG00000061100	264	69	-1.939	3.46E-12	Retnla
ENSMUSG00000027931	1212	2185	0.851	3.48E-12	Npr1
ENSMUSG00000008393	4940	2852	-0.793	3.56E-12	Carhsp1
ENSMUSG00000079012	13	67	2.360	3.81E-12	Serpina3m
ENSMUSG00000022485	362	155	-1.226	3.81E-12	Hoxc5
ENSMUSG00000023055	2421	4250	0.812	3.91E-12	Calcoco1
ENSMUSG00000006301	1979	5322	1.427	3.92E-12	Tmbim1
ENSMUSG00000018076	1057	1591	0.590	4.19E-12	Med13l
ENSMUSG00000039270	1413	706	-1.000	4.21E-12	Megf9
ENSMUSG00000086290	233	502	1.102	4.40E-12	Snhg12
ENSMUSG00000040213	1361	2469	0.859	4.69E-12	Kyat3
ENSMUSG00000028138	2207	3785	0.778	4.72E-12	Adh5
ENSMUSG00000017167	1307	464	-1.496	4.72E-12	Cntnap1
ENSMUSG00000022814	329	951	1.533	5.23E-12	Umps
ENSMUSG00000022946	661	1313	0.991	6.30E-12	Dopey2
ENSMUSG00000021273	3555	10589	1.574	6.71E-12	Fdft1
ENSMUSG00000020387	276	530	0.942	6.90E-12	Jade2
ENSMUSG00000026193	764	273	-1.485	7.07E-12	Fn1
ENSMUSG00000003500	6525	18844	1.530	7.52E-12	Impdh1
ENSMUSG00000030246	13342	19977	0.582	8.38E-12	Ldhb
ENSMUSG00000060568	200	63	-1.669	8.80E-12	Fam78b
ENSMUSG00000064337	66526	40945	-0.700	8.84E-12	mt-Rnr1
ENSMUSG00000022871	37	179	2.284	9.11E-12	Fetub
ENSMUSG00000028359	466	209	-1.156	9.11E-12	Orm3
ENSMUSG00000024424	51	248	2.290	9.15E-12	Ttc39c
ENSMUSG00000024713	466	1670	1.840	9.15E-12	Pcsk5
ENSMUSG00000031023	303	576	0.923	9.28E-12	Akip1

ENSMUSG00000024065	164	550	1.750	9.86E-12	Ehd3
ENSMUSG00000028076	4483	2492	-0.847	1.07E-11	Cd1d1
ENSMUSG00000032193	423	96	-2.139	1.14E-11	Ldlr
ENSMUSG00000011148	1183	2795	1.241	1.21E-11	Adssl1
ENSMUSG00000028838	14	107	2.877	1.29E-11	Extl1
ENSMUSG00000004040	1833	3575	0.963	1.34E-11	Stat3
ENSMUSG00000026582	47	214	2.171	1.34E-11	Sele
ENSMUSG00000022426	2240	3850	0.782	1.44E-11	Josd1
ENSMUSG00000032092	688	1290	0.907	1.48E-11	Mpzl2
ENSMUSG00000036446	438	97	-2.175	1.60E-11	Lum
ENSMUSG00000029190	2715	1588	-0.774	1.62E-11	D5Ertd579e
ENSMUSG00000038624	111	33	-1.750	1.66E-11	Nepn
ENSMUSG00000073433	63	219	1.806	1.68E-11	Arhgdig
ENSMUSG00000030546	33532	14540	-1.206	1.74E-11	Plin1
ENSMUSG00000055799	244	537	1.140	1.87E-11	Tcf7l1
ENSMUSG00000029416	340	709	1.062	1.87E-11	Slc15a4
ENSMUSG00000058486	594	1571	1.402	2.05E-11	Wdr91
ENSMUSG00000031167	1627	2930	0.849	2.15E-11	Rbm3
ENSMUSG00000019278	782	1375	0.815	2.22E-11	Dpep1
ENSMUSG00000039886	3674	1724	-1.092	2.35E-11	Tmem120a
ENSMUSG00000062867	487	904	0.893	2.37E-11	Impdh2
ENSMUSG00000044338	238	52	-2.198	2.41E-11	Aplnr
ENSMUSG00000038520	974	1620	0.734	2.47E-11	Tbc1d17
ENSMUSG00000041775	745	1834	1.299	2.49E-11	Mapk1ip1
ENSMUSG00000029716	139	26	-2.425	2.95E-11	Tfr2
ENSMUSG00000042129	456	1420	1.638	3.53E-11	Rassf4
ENSMUSG00000037104	655	382	-0.778	3.62E-11	Socs5
ENSMUSG00000036304	81	335	2.034	3.79E-11	Zdhhc23
ENSMUSG00000030495	464	262	-0.824	3.79E-11	Slc7a10
ENSMUSG00000022564	8593	16724	0.961	3.87E-11	Grina
ENSMUSG00000026313	261	628	1.266	4.01E-11	Hdac4
ENSMUSG00000013846	1427	2809	0.977	4.01E-11	St3gal1
ENSMUSG00000023078	82	438	2.420	4.10E-11	Cxcl13
ENSMUSG00000027075	365	1026	1.490	4.23E-11	Slc43a1
ENSMUSG00000045620	205	51	-2.004	4.26E-11	Odf3l1
ENSMUSG00000028274	476	257	-0.893	4.29E-11	Rngtt
ENSMUSG00000079037	2188	1129	-0.955	4.73E-11	Prnp
ENSMUSG00000045573	33	115	1.810	4.88E-11	Penk
ENSMUSG00000003534	246	109	-1.169	5.22E-11	Ddr1
ENSMUSG00000057421	1166	2094	0.845	5.52E-11	Las1l
ENSMUSG00000034235	162	467	1.522	5.95E-11	Usp54
ENSMUSG00000033161	2187	3674	0.749	6.11E-11	Atp1a1
ENSMUSG00000030126	481	1287	1.417	6.12E-11	Tmcc1
ENSMUSG00000050737	269	99	-1.444	6.32E-11	Ptges
ENSMUSG00000026888	746	267	-1.483	6.32E-11	Grb14
ENSMUSG00000050390	184	408	1.151	6.66E-11	C77080
ENSMUSG00000002103	1153	742	-0.637	6.70E-11	Acp2
ENSMUSG00000005667	143	589	2.038	6.78E-11	Mthfd2
ENSMUSG00000024998	230	87	-1.407	7.02E-11	Plce1
ENSMUSG00000002769	126	390	1.630	7.28E-11	Gnmt
ENSMUSG00000068686	265	121	-1.130	7.28E-11	Cd59b
ENSMUSG00000001348	3261	1858	-0.812	7.28E-11	Acp5

ENSMUSG00000027762	61	11	-2.520	7.77E-11	Sucnr1
ENSMUSG00000001119	2362	933	-1.339	8.28E-11	Col6a1
ENSMUSG00000020780	1028	1600	0.639	8.46E-11	Srp68
ENSMUSG00000029475	238	441	0.890	8.70E-11	Kdm2b
ENSMUSG00000032911	2134	773	-1.464	8.88E-11	Cspg4
ENSMUSG00000034981	6287	2164	-1.538	9.48E-11	Parm1
ENSMUSG00000078515	1484	2488	0.745	1.01E-10	Ddi2
ENSMUSG00000021477	3139	5220	0.734	1.03E-10	Ctsl
ENSMUSG00000012114	835	1310	0.649	1.05E-10	Med15
ENSMUSG00000071637	239	813	1.764	1.12E-10	Cebpd
ENSMUSG00000052957	1062	2758	1.377	1.20E-10	Gas1
ENSMUSG00000028245	879	1501	0.772	1.25E-10	Nsmaf
ENSMUSG00000042246	36	132	1.888	1.25E-10	Tmc7
ENSMUSG00000026380	10	84	3.049	1.33E-10	Tfcp2l1
ENSMUSG00000042429	2451	1400	-0.809	1.33E-10	Adora1
ENSMUSG00000020448	863	1545	0.840	1.36E-10	Rnf185
ENSMUSG00000041075	622	306	-1.026	1.37E-10	Fzd7
ENSMUSG00000041313	94	256	1.443	1.46E-10	Slc7a1
ENSMUSG00000041679	100	222	1.149	1.57E-10	Lrrc29
ENSMUSG00000037697	1213	2310	0.929	1.59E-10	Ddhd1
ENSMUSG00000022453	662	1090	0.721	1.87E-10	Naga
ENSMUSG00000044018	1955	1304	-0.584	1.94E-10	Mrpl50
ENSMUSG00000021094	4146	2552	-0.700	2.15E-10	Dhrs7
ENSMUSG00000017386	1783	718	-1.312	2.15E-10	Traf4
ENSMUSG00000032350	709	1263	0.832	2.18E-10	Gclc
ENSMUSG00000026621	324	1152	1.832	2.20E-10	44256
ENSMUSG00000034832	910	564	-0.690	2.29E-10	Tet3
ENSMUSG00000027221	467	204	-1.197	2.36E-10	Chst1
ENSMUSG00000059248	3409	1125	-1.600	2.45E-10	44448
ENSMUSG00000050549	279	138	-1.022	2.51E-10	Fam241a
ENSMUSG00000035104	119	395	1.729	2.54E-10	Eva1a
ENSMUSG00000025509	64807	120008	0.889	2.59E-10	Pnpla2
ENSMUSG00000017707	6983	11691	0.743	2.62E-10	Serinc3
ENSMUSG00000026005	1859	1192	-0.641	2.72E-10	Rpe
ENSMUSG00000041592	190	578	1.604	2.87E-10	Sdk2
ENSMUSG00000048911	606	253	-1.261	2.94E-10	Rnf24
ENSMUSG00000028341	458	3303	2.851	3.32E-10	Nr4a3
ENSMUSG00000000901	61	160	1.397	3.57E-10	Mmp11
ENSMUSG00000015837	6135	11595	0.918	3.62E-10	Sqstm1
ENSMUSG00000021270	6113	2511	-1.284	3.66E-10	Hsp90aa1
ENSMUSG00000003477	185	924	2.317	3.85E-10	Inmt
ENSMUSG00000029022	365	651	0.834	3.91E-10	Miip
ENSMUSG00000037461	686	376	-0.865	3.97E-10	Ints7
ENSMUSG00000006920	1152	1828	0.667	4.47E-10	Ezh1
ENSMUSG00000026279	296	509	0.782	4.80E-10	Thap4
ENSMUSG00000085795	2946	1399	-1.074	4.87E-10	Zfp703
ENSMUSG00000004043	1785	953	-0.905	5.05E-10	Stat5a
ENSMUSG00000072825	212	442	1.059	5.22E-10	Cep170b
ENSMUSG00000033159	1234	2006	0.700	5.43E-10	Cnppd1
ENSMUSG00000095789	85	233	1.452	5.63E-10	Nupr1l
ENSMUSG00000029017	4917	3444	-0.514	5.79E-10	Pmpcb
ENSMUSG00000034158	3008	5726	0.929	5.87E-10	Lrrc58

ENSMUSG00000040462	1907	2670	0.486	6.17E-10	Os9
ENSMUSG00000026819	212	1634	2.945	6.38E-10	Slc25a25
ENSMUSG00000042677	302	128	-1.242	6.74E-10	Zc3h12a
ENSMUSG00000009633	8420	556	-3.920	7.83E-10	G0s2
ENSMUSG00000024039	16	115	2.870	8.12E-10	Cbs
ENSMUSG00000015536	2328	3634	0.642	8.13E-10	Mocs2
ENSMUSG00000027901	163	386	1.247	8.65E-10	Dennd2d
ENSMUSG00000031373	2067	4052	0.971	8.68E-10	Car5b
ENSMUSG00000028207	1292	3587	1.473	8.96E-10	Asph
ENSMUSG00000022324	298	120	-1.312	8.96E-10	Matn2
ENSMUSG00000022637	860	2656	1.627	9.51E-10	Cblb
ENSMUSG00000056427	284	539	0.925	1.01E-09	Slit3
ENSMUSG00000042834	402	122	-1.726	1.05E-09	Nrep
ENSMUSG00000020019	118	269	1.193	1.07E-09	Ntn4
ENSMUSG00000034424	2625	1796	-0.548	1.31E-09	Gcsh
ENSMUSG00000032334	619	218	-1.508	1.32E-09	Loxl1
ENSMUSG00000000594	2144	1464	-0.551	1.37E-09	Gm2a
ENSMUSG00000042540	22	142	2.731	1.50E-09	Acot5
ENSMUSG00000028085	1266	783	-0.694	1.52E-09	Gatb
ENSMUSG00000019437	1904	1068	-0.834	1.53E-09	Tlcd1
ENSMUSG00000007682	6073	902	-2.751	1.53E-09	Dio2
ENSMUSG00000024968	97	25	-1.976	1.68E-09	Rcor2
ENSMUSG00000041264	638	1038	0.703	1.69E-09	Uspl1
ENSMUSG00000046756	2466	1619	-0.607	1.73E-09	Mrps7
ENSMUSG00000053886	107	352	1.721	1.79E-09	Sh2d4a
ENSMUSG00000047182	453	150	-1.592	1.79E-09	Irs3
ENSMUSG00000022948	310	558	0.847	2.00E-09	Setd4
ENSMUSG00000018572	664	327	-1.020	2.08E-09	Phf23
ENSMUSG00000029405	1820	1070	-0.767	2.19E-09	G3bp2
ENSMUSG00000038984	25	97	1.936	2.28E-09	Tspyl5
ENSMUSG00000026767	694	1103	0.667	2.32E-09	Mindy3
ENSMUSG00000035944	1218	2057	0.756	2.38E-09	Ttc38
ENSMUSG00000041124	681	426	-0.677	2.41E-09	Msantd4
ENSMUSG00000020072	77	168	1.129	2.50E-09	Pbld2
ENSMUSG00000074364	8179	4049	-1.014	2.57E-09	Ehd2
ENSMUSG00000027559	103392	36663	-1.496	2.91E-09	Car3
ENSMUSG00000030088	2021	3665	0.859	3.06E-09	Aldh1l1
ENSMUSG00000066798	377	216	-0.805	3.14E-09	Zbtb6
ENSMUSG00000028042	3935	1753	-1.166	3.19E-09	Zbtb7b
ENSMUSG00000028822	1933	2987	0.628	3.31E-09	Tmem50a
ENSMUSG00000047246	114	24	-2.230	3.37E-09	Hist1h2be
ENSMUSG00000025372	273	692	1.344	4.24E-09	Baiap2
ENSMUSG00000006127	872	1306	0.584	4.36E-09	Inpp5k
ENSMUSG00000046167	114	27	-2.077	4.38E-09	Gldn
ENSMUSG00000025059	7878	3994	-0.980	4.46E-09	Gk
ENSMUSG00000020788	380	795	1.067	4.49E-09	Atp2a3
ENSMUSG00000056133	23	129	2.440	4.55E-09	Unc93a2
ENSMUSG00000001082	216	396	0.873	4.73E-09	Mfsd10
ENSMUSG00000039137	78	164	1.086	4.84E-09	Whrn
ENSMUSG00000026509	4067	2302	-0.821	5.21E-09	Capn2
ENSMUSG00000006931	237	446	0.912	5.27E-09	P3h4
ENSMUSG00000029466	811	1289	0.669	5.27E-09	Anapc7

ENSMUSG00000026791	543	836	0.623	5.27E-09	Slc2a8
ENSMUSG00000038342	1316	2190	0.735	5.33E-09	Mlxip
ENSMUSG00000000204	363	7	-5.762	5.40E-09	Slfn4
ENSMUSG00000024413	1988	4284	1.108	5.40E-09	Npc1
ENSMUSG00000021681	1260	770	-0.710	5.40E-09	Aggf1
ENSMUSG00000032633	5018	9450	0.913	5.50E-09	Flcn
ENSMUSG00000036186	494	162	-1.609	5.74E-09	Fam69b
ENSMUSG00000034430	303	495	0.709	5.89E-09	Zxdc
ENSMUSG00000054312	2339	1490	-0.651	5.96E-09	Mrps21
ENSMUSG00000045502	186	86	-1.115	5.96E-09	Hcar2
ENSMUSG00000032594	2022	3100	0.617	6.45E-09	Ip6k1
ENSMUSG00000030714	256	415	0.697	6.51E-09	Sgf29
ENSMUSG00000037493	7058	3102	-1.186	6.60E-09	Cib2
ENSMUSG00000030707	333	86	-1.947	6.67E-09	Coro1a
ENSMUSG00000023886	618	308	-1.002	6.79E-09	Smoc2
ENSMUSG00000035279	156	48	-1.687	6.86E-09	Ssc5d
ENSMUSG00000043154	801	2080	1.376	6.89E-09	Ppp2r3a
ENSMUSG00000024949	3077	5117	0.734	6.89E-09	Sf1
ENSMUSG00000008429	884	1312	0.570	7.21E-09	Herpud2
ENSMUSG00000023960	1122	1806	0.686	7.25E-09	Enpp5
ENSMUSG00000020914	66	5	-3.675	7.25E-09	Top2a
ENSMUSG00000007476	317	570	0.846	7.66E-09	Lrrc8a
ENSMUSG00000020715	435	831	0.935	7.87E-09	Ern1
ENSMUSG00000032715	201	730	1.860	8.33E-09	Trib3
ENSMUSG00000068036	1910	1154	-0.728	8.95E-09	Afdn
ENSMUSG00000054555	3856	1909	-1.014	9.58E-09	Adam12
ENSMUSG00000026042	920	379	-1.278	9.86E-09	Col5a2
ENSMUSG00000029804	751	1492	0.990	1.07E-08	Herc3
ENSMUSG00000018326	8449	5773	-0.550	1.08E-08	Ywhab
ENSMUSG00000075232	706	430	-0.717	1.13E-08	Amd1
ENSMUSG00000029512	1913	3387	0.824	1.13E-08	Ulk1
ENSMUSG00000007655	14608	9294	-0.653	1.15E-08	Cav1
ENSMUSG00000001983	1014	674	-0.590	1.19E-08	Taco1
